# A network pharmacology based approach for predicting active ingredients and potential mechanism of Lianhuaqingwen capsule in treating COVID-19

**DOI:** 10.7150/ijms.53685

**Published:** 2021-02-24

**Authors:** Xiaobo Zhang, Rui Gao, Zubing Zhou, Xuehua Tang, Jingjing Lin, Long Wang, Xin Zhou, Tao Shen

**Affiliations:** 1 School of Basic Medicine, Chengdu University of Traditional Chinese Medicine, Chengdu, China.; 2 Academic Department, Zhuhai Ebang Pharmaceutical Co., Ltd. Zhuhai, China.

**Keywords:** Lianhuaqingwen capsule, COVID-19, 3CL pro, SARS-CoV-2, network pharmacology

## Abstract

The outbreak of severe respiratory disease caused by SARS-CoV-2 has led to millions of infections and raised global health concerns. Lianhuaqingwen capsule (LHQW-C), a traditional Chinese medicine (TCM) formula widely used for respiratory diseases, shows therapeutic efficacy in the application of coronavirus disease 2019 (COVID-19). However, the active ingredients, drug targets, and the therapeutic mechanisms of LHQW-C in treating COVID-19 are poorly understood. In this study, an integrating network pharmacology approach including pharmacokinetic screening, target prediction (targets of the host and targets from the SARS-CoV-2), network analysis, GO enrichment analysis, KEGG pathway enrichment analysis, and virtual docking were conducted. Finally, 158 active ingredients in LHQW-C were screen out, and 49 targets were predicted. GO function analysis revealed that these targets were associated with inflammatory response, oxidative stress reaction, and other biological processes. KEGG enrichment analysis indicated that the targets of LHQW-C were highly enriched to several immune response-related and inflammation-related pathways, including the IL-17 signaling pathway, TNF signaling pathway, NF-kappa B signaling pathway, and Th17 cell differentiation. Moreover, four key components (quercetin, luteolin, wogonin, and kaempferol) showed a high binding affinity with SARS-CoV-2 3-chymotrypsin-like protease (3CL pro). The study indicates that some anti-inflammatory ingredients in LHQW-C probably modulate the inflammatory response in severely ill patients with COVID-19.

## Introduction

Coronaviruses are enveloped RNA viruses, of which the pandemic causes a serious threat to humans and evolved into public health events 1, 2. In December 2019, a severe respiratory disease (COVID-19) was identified in China, which is caused by a new virus, SARS-CoV-2. As of 14 April 2020, over 1,700,000 cases were confirmed with 100,000 deaths over 100 countries worldwide 3, 4. Among these COVID-19 patients, common symptoms include fever, cough, myalgia, and dyspnea 5-7. More seriously, infected individuals can develop pneumonia, acute respiratory distress syndrome (ARDS), multiorgan failure, and even death 8, 9. So far, no vaccines or specific drugs have been found for COVID-19, and the primary treatments are supportive.

Lianhuaqingwen capsule (LHQW-C), a Chinese patent medicine contains 13 medicinal herbs ( Fructus Forsythiae, Flos Lonicerae Japonicae, Herba Ephedrae, Almond, Radix Isatidis, Fortunes Boss fern Rhizome, Herba Houttuyniae, Herba Pogostemonis, Rheum palmatum, Rhodiola rosea, Glycyrrhiza uralensis Fisch, Mentha haplocalyx Briq and Gypsum Fibrosum) is derived from two classics prescriptions: Ma Xing Shi Gan Tang (MXSGT) and Yin Qiao San (YQS). MXSGT, a notable classic formula recorded in Shanghan Lun edited by Zhang Zhongjing in Han Dynasty, is mainly prescribed for the treatments of febrile diseases, including influenza, acute bronchitis, and pneumonia 10, 11. YQS is a famous prescription of Dr. Wu Jutong in Qing dynasty, mainly prescribed in the treatment of colds, pneumonia. Among the Diagnosis and Treatment of Novel Coronavirus Pneumonia (Version 4/Version 5/Version 6/Version 7) issued by China's National Health Commission, LHQW-C was recommended as one of the basic TCM for the treatment of COVID-19.

**Figure 1:**
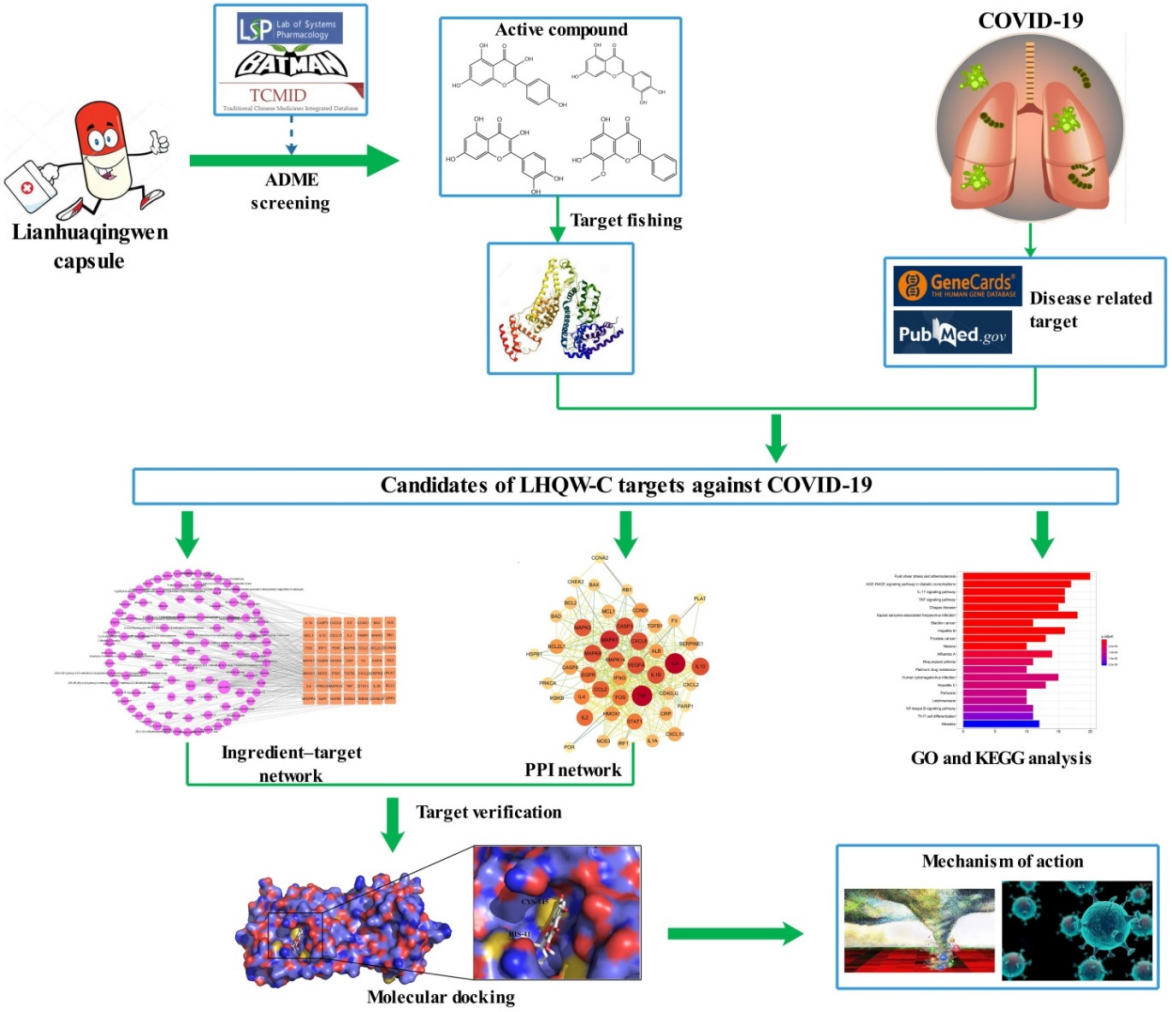
Flow chart of the network pharmacology based study.

A retrospective and observational study demonstrated that LHQW-C could improve patient clinical symptoms, including fever, cough, chest tightness, and decreased appetite 12. Additionally, according to a multicenter, prospective, randomized controlled trial for patients with COVID-19, LHQW-C could ameliorate the efficiency of clinical treatment, shorten the time of fever, cough, and fatigue, improve chest CT findings, and accelerate the recovery of COVID-19 patients 13. However, little is known about the active ingredients, drug targets, and the therapeutic mechanisms of LHQW-C in treating COVID-19, traditional method may be hard to understand how such a complicate prescription exert their biological effects through multiple targets and synergistic effects on human systems. Network pharmacology follows “network target, multicomponent therapeutics” model, shifting the paradigm from the concept of one gene, one target, and one disease 14, 15. It provides a novel concept for comprehending the multitargeted mechanism of the treatment of sophisticated diseases such as COVID-19 16. In this study, a systematic pharmacological methodology was applied to explore the important material basis and the underlying biological mechanism of LHQW-C in COVID-19 treatment. Considering that many TCM prescriptions have been reported to inhibit the replication of the virus directly, we also examined the effect of the ingredients of LHQW-C on virus suppression in our study. The flow chart of the study is shown in Figure 1.

## Materials and Methods

### Chemical database construction

LHQW-C was produced by Yiling Pharmaceutical Co. Ltd. (Shijiazhuang, China), which composed of 13 herbs, including Fructus Forsythiae, Flos Lonicerae Japonicae, Herba Ephedrae, Almond, Radix Isatidis, Fortunes Boss fern Rhizome, Herba Houttuyniae, Herba Pogostemonis, Rheum palmatum, Rhodiola rosea, Glycyrrhiza uralensis Fisch, Mentha haplocalyx Briq and Gypsum Fibrosum. All ingredients of LHQW-C were retrieved from the natural product databases for Chinese herbal medicines: Traditional Chinese Medicine Systems Pharmacology (TCMSP) database 17. Oral bioavailability (OB) and drug-likeness (DL) are two important pharmacokinetic parameters. Components with OB ≥30% and DL ≥0.18 are more likely to be drugs. Hence these two parameters were set as criteria to select candidate ingredients in TCMSP 18, 19. However, some herbs can't be found in the database, such as Rhodiola rosea, Mentha and Gypsum, the three herbs were retrieved using BATMAN-TCM and TCMID databases 20, 21. Since the protein name in TCMSP is not standard, we used UniProtKB to obtain their official symbols 22.

### COVID-19 related targets

Genecards was utilized to acquire COVID-19 related genes, which were the targets of the host 23. “COVID-19”, “Sars-Cov-2” and “novel sars-cov” were used as keywords to search for genes that may be associated with this novel disease. The results were combined and duplicate genes were removed. Whereas Genecards mainly provides information on human genes, we therefore consulted PubMed to search the primary targets for virus replication.

### Drug-disease common genes

A Venn diagram was drawn to visualize the intersected genes between the active ingredients and COVID-19 24.

### Protein-protein interaction (PPI)

Proteins seldom act alone, they usually realize the biological functions through interactions with other proteins. Therefore, the String database was employed to predict PPI interaction data 25. The common genes obtained were searched in the String database using the multiple proteins option, the species was limited to “Homo sapiens”, and a confidence score >0.7 was set as the cut-off criterion 26. The TSV format of the result was download for further Ingredient-disease PPI network construction.

### GO and KEGG pathway enrichment analysis

To determine biological processes and molecular interactions associated with selected common genes, Gene Ontology (GO) enrichment and Kyoto Encyclopedia of Genes and Genomes (KEGG) 27 pathway enrichment were carried out using R version 3.6.3, the top 20 terms with p-value <0.01 were selected.

### Network construction

Network construction and analysis were performed using Cytoscape software (version 3.7.2) 28. Two networks were constructed: (1) Ingredient-target network; (2) Ingredient-disease PPI network. The network is composed of nodes and edges. Each node in the network represents a molecule (ingredient or target), and each edge represents a biological relationship between two nodes.

### Molecular docking

To validate the ingredient-target association and evaluate the effect of LHQW-C on virus suppression in our study, we used AutoDock Vina (version 1.1.2) for virtual docking 29. The target protein three-dimensional (3D) structures were obtained from the PDB database and processed using the PyMol software (version 1.7.2.1) 30, 31, including removing the ligands, correcting protein structure, and removing water. The proteins and ligands were converted to the PDBQT format in the AutoDockTools (version1.5.6) prior to performing the docking process 32, 33. Because the active side of 3CL pro is centered on the amino acid residues of the original ligand, the “active pocket” was constructed as follows: grid center -10.817, 11.746, 68.213 and NPTS 42, 60, 42, 0.375. Similar docking conditions were used and the Lamarckian genetic algorithm was chosen. Affinity binding results <0 kcal/mol indicates that the ligand can bind to the receptor, affinity binding results ≤-5.0 kcal/mol show a better binding ability. The docking results were visualized by PyMol and Discovery Studio 2016 (version 16.1).

## Results

### Ingredient-target network

**Figure 2:**
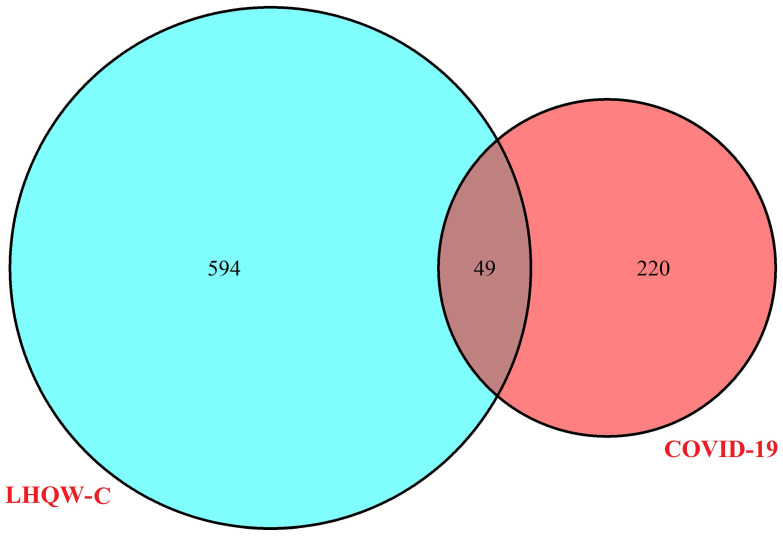
The 49 overlapping genes between COVID-19 and LHQW-C.

263 ingredients of the 13 herbs of LHQW-C were collected from the public databases (TCMSP, BATMAN, and TCMID) (Supplementary Table 1). The duplicated portions were removed and 226 compounds were retained for further study (Supplementary Table 2). 269 active ingredients related targets were screened out after the elimination of repetitive component-targets (Supplementary Table 3). 643 therapeutic genes for COVID-19 were collected from GeneCards (Supplementary Table 4). Intersected genes were generated based on the overlap between LHQW-C and COVID-19, and 49 targets were common to “drug-disease” (Figure 2), the details are described in (Supplementary Table 5). This means that these 49 genes may be the key of LHQW-C in treating COVID-19.

**Figure 3:**
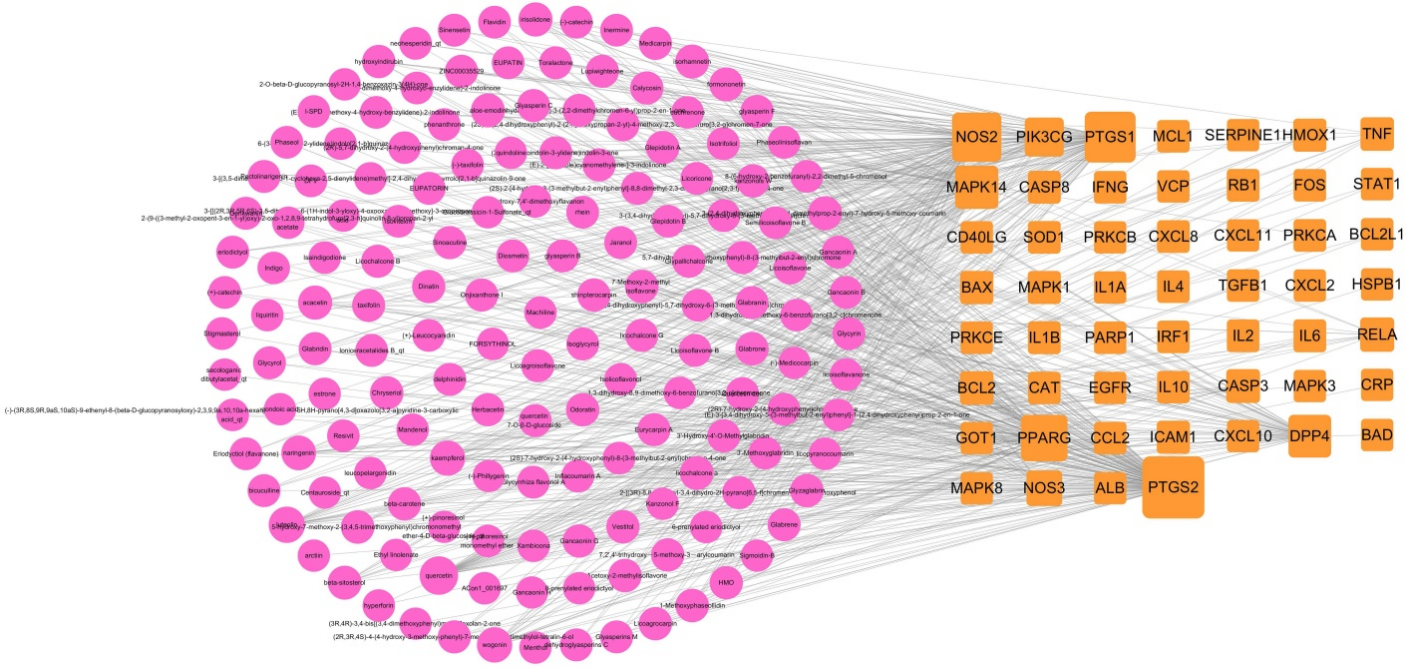
Ingredient-target network of LHQW-C. The red node represents ingredient, the orange node represents target.

The ingredient-target network comprised 153 (49 targets and 104 compounds) nodes and 299 edges (Figure 3). As shown in this figure, some special compounds displayed more intimate association to multiple targets, playing a major role in network regulation, such as quercetin (degree 38), luteolin (degree 17), wogonin (degree 12), and kaempferol (degree 11). This suggests that LHQW-C may exert synergistic pharmacological effects on COVID-19.

### Ingredient-COVID-19 PPI network

**Figure 4:**
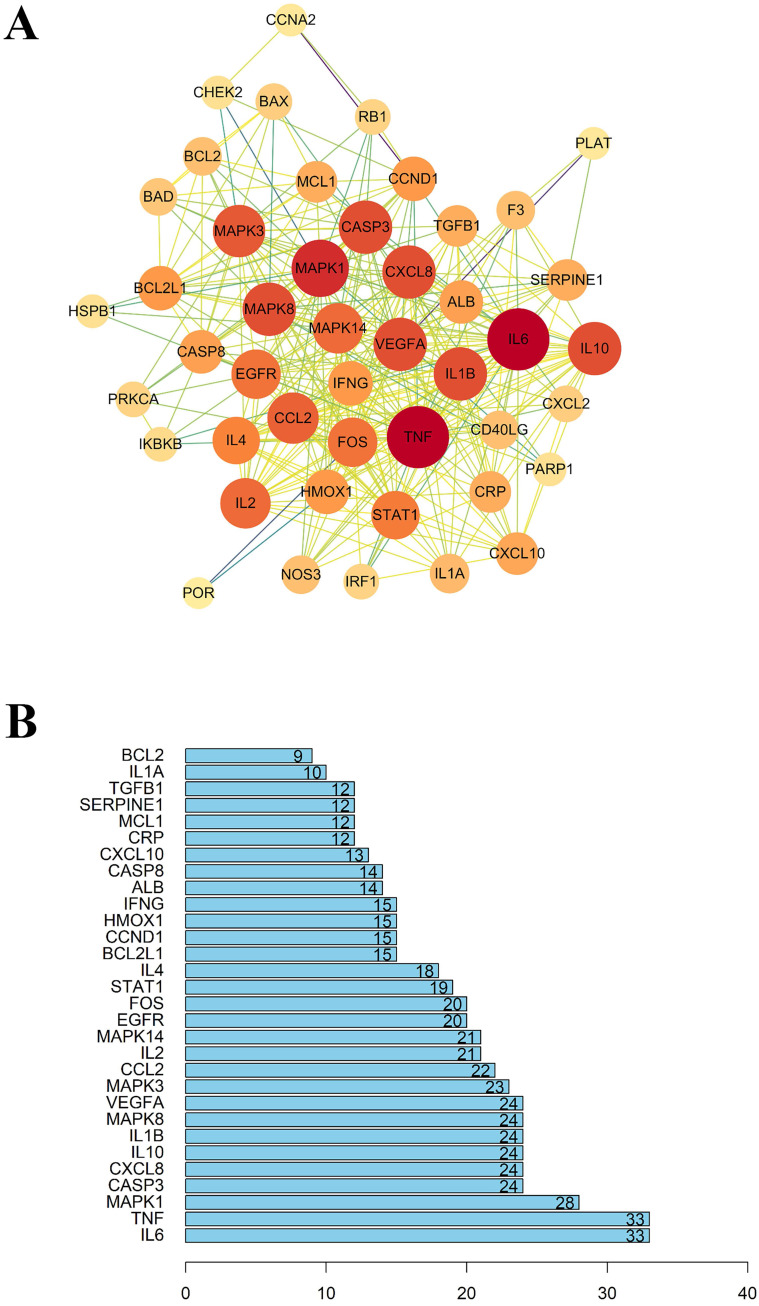
(A) The protein-protein interaction (PPI) network. (B) The bar plot of the protein-protein interaction (PPI) network. The X-axis represents the number of neighboring proteins of the target protein. The Y-axis represents the target proteins.

As mentioned above, 49 intersected genes were obtained. Ingredient-disease co-genes were imported into STRING, and we obtained the PPI network with the medium degree of confidence level (degree > 0.7). As shown in Figure 4, the PPI network consisted of 46 nodes and 331 edges. 'Degree' represents the number of links to node, 'Betweenness Centrality' is used to measure how often a node lies on the shortest path between nodes in the network, 'Closeness Centrality' represents the distance between the individuals and all other peers in a network, these three parameters are used to measure the importance of the proteins in the PPI network 34, 35. Therefore, nodes with an average value of degree ≥27, node betweenness ≥0.0009993, and closeness ≥0.618 could be considered as hubs, including IL-6, TNF, MAPK1, these targets tended to execute more critical roles in the target network of LHQW-C.

### GO functional enrichment analysis

**Figure 5:**
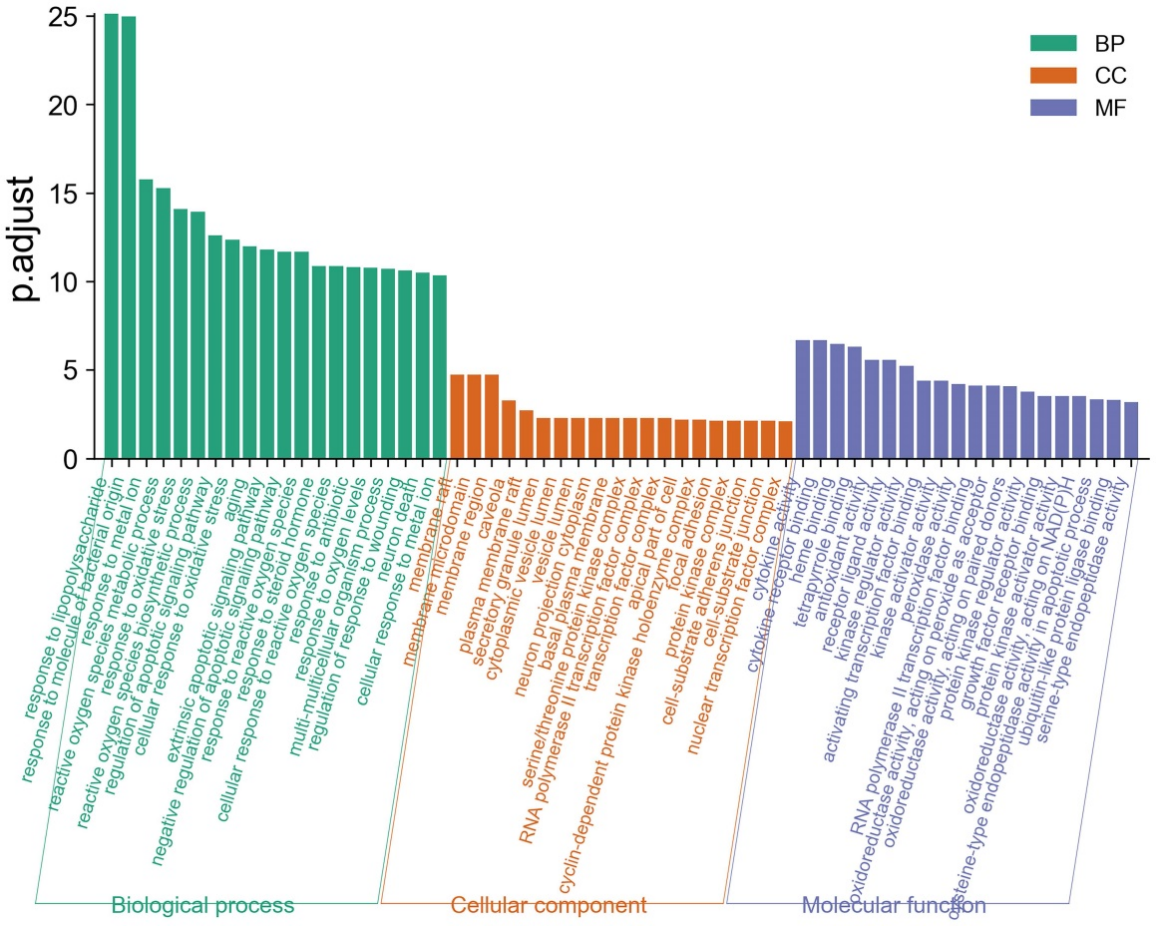
Gene Ontology (GO) analysis of the 49 overlapping gene symbols associated with COVID-19. The X-axis represents the categories in the GO of the target genes, while the Y-axis represents the P.adjust (-log10) in the GO of the target genes.

GO enrichment analysis describes a complex cascade of events, which is accomplished by one or more components through well-organized molecular interactions 36. We displayed the top 20 biological process (BP), molecular function (MF), and cellular component (CC) terms in Figure 5, the details of the GO analysis are described in Supplementary Table 6. In the BP category, LHQW-C was significantly enriched to response to lipopolysaccharide, response to molecule of bacterial origin, response to metal ion, reactive oxygen species metabolic process, response to oxidative stress, reactive oxygen species biosynthetic process, regulation of apoptotic signaling pathway, cellular response to oxidative stress, and so forth. These results help to understand the biological function changes in the body after treatment with LHQW-C.

### KEGG pathway enrichment analysis

**Figure 6:**
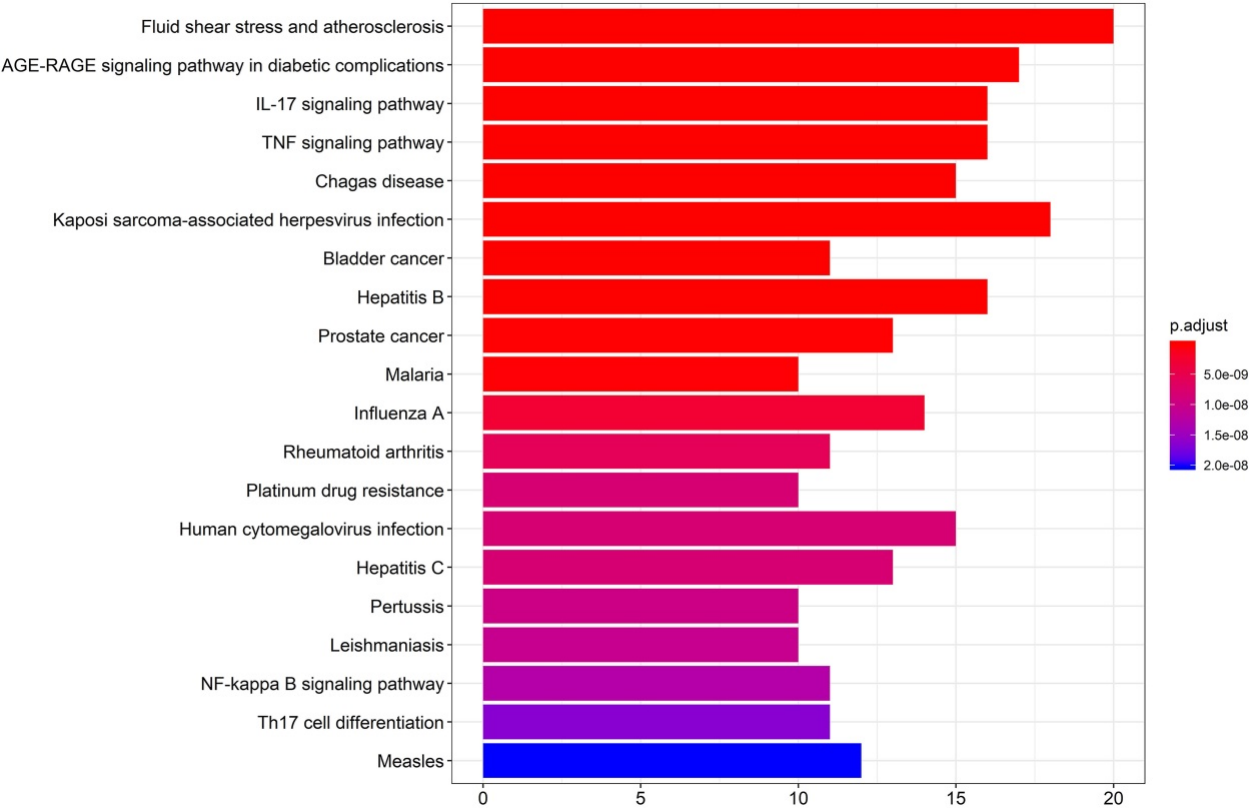
The Kyoto Encyclopedia of Genes and Genomes (KEGG) pathway enrichment analysis. The X-axis represents the target counts in each pathway, and the Y-axis represents the main pathways (P-value < 0.01).

KEGG pathway enrichment was performed by R version 3.6.3 (Figure 6). The first 20 pathways were shown according to the p-value from small to large, the details are described in Supplementary Table 7. As described in the figure, the 49 “drug-disease” genes were highly related to several immune response-related and inflammation-related pathways, including the IL-17 signaling pathway, TNF signaling pathway, NF-kappa B signaling pathway, and Th17 cell differentiation.

### Molecular docking

**Figure 7:**
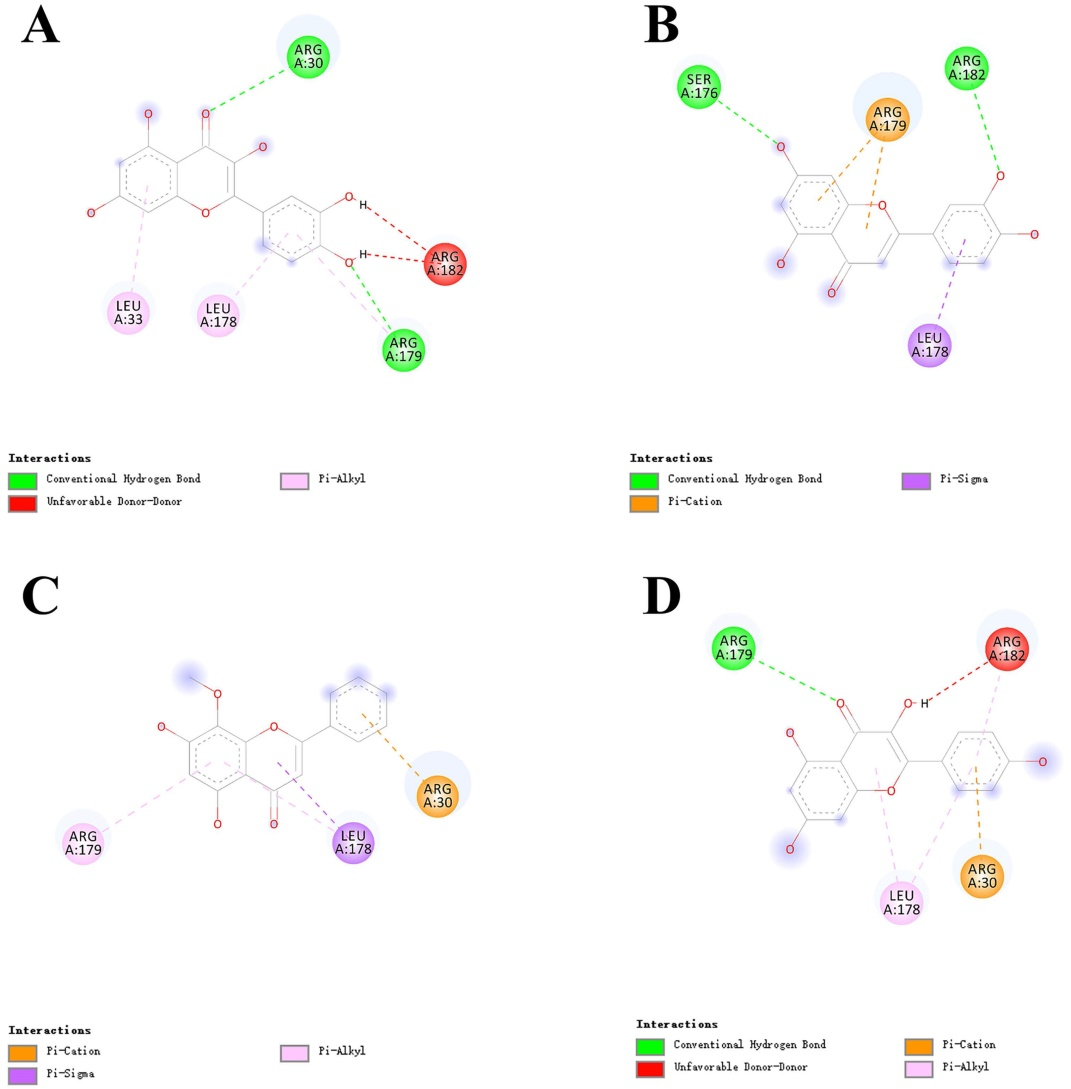
Virtual docking of bioactive ingredients from LHQW-C with key therapeutic targets. (A) quercetin with IL-6. (B) luteolin with IL-6. (C) wogonin with IL-6. (D) kaempferol with IL-6.

**Figure 8:**
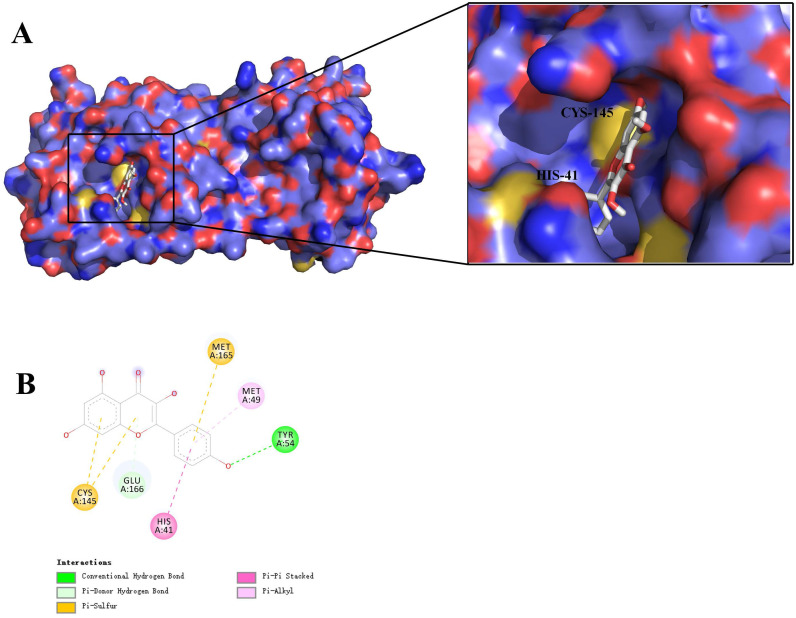
Virtual docking of key ingredients with 3CL pro. (A) 3D docking conformation of kaempferol with 3CL pro; (B) 2D docking conformation of kaempferol with 3CL pro.

To further assess the interaction and improve the accuracy between the active ingredients and their targets, 4 active ingredients (quercetin, luteolin, wogonin, kaempferol,) and 9 targets (IL-6, TNF, MAPK1, CASP3, CXCL8, IL-10, IL1B, MAPK8, and VEGFA) were selected for docking. In addition, we simultaneously chose 3CL pro for another docking to investigate whether LHQW-C has an effect on SARS-CoV-2. Before docking, the three-dimensional (3D) structures of the proteins 1alu, 5uui, 3w55, 1re1, 3il8, 4x51, 2nvh, 4g1w, 4kzn and 6lu7 were obtained by searching IL-6 , TNF, MAPK1, CASP3, CXCL8, IL-10, IL1B, MAPK8, VEGFA and 3CL pro from the PDB protein database, respectively. The docking scores were depicted in Table 1 and Table 2, and the docking conformation was shown in Figure 7 and Figure 8, respectively. Compounds with lower binding energies are usually considered to exhibit a higher binding affinity with the target protein.

As shown in Figure 6, the 4 key ingredients were successfully docked to target IL-6, the interactions between the active site residues and the ligands include hydrogen bonding, π-cation, π-alkyl, and π-sigma. Moreover, these ingredients were fitted well in the active pocket of 3CL pro and showed a high binding affinity with it. In Figure 7, kaempferol bounds at the active site of the enzyme and interacted with the key residues (Cys145 and His41), and other residues, such as GLU166, MET165, MET49, and TYR54. This suggests that these compounds might be the potential inhibitors when LHQW-C treats SARS-CoV-2.

## Discussion

Since December 2019, a severe respiratory disease caused by a new coronavirus has swept the mainland of China and then discovered in over hundreds of countries and regions worldwide 37. The global public health problem caused by the ongoing SARS-CoV-2 epidemic, making researchers pay attention to finding broad-spectrum antiviral agents which may help reduce the detrimental effects of human coronavirus infection. However, further validations *in vivo* are required to demonstrate the efficacy and safety of novel candidates, even some approved drugs, which means that it will consume more time for clinical practices. Up to now, there is no specific drugs for the treatment of COVID-19, the therapeutic strategies are still very limited 38. Fortunately, the multi-ingredients, multi-targets synergistic action of TCM may be beneficial against COVID-19 39, 40. As a common TCM prescription for respiratory diseases, LHQW-C has been used extensively for more than 10 years in China. It has curative effects on acute bronchitis, asthma, SARS, and influenza 41. After the outbreak of COVID-19, LHQW-C was recommended as one of the fundamental drugs in the *Guideline for the Diagnosis and Treatment of Novel Coronavirus (2019-nCoV)*. In this study, network pharmacology was applied to explore the latent mechanism of LHQW-C in COVID-19 treatment.

It was classically considered that virus surmounts the host defense and cause disease in some cases 42, 43. Fatal pneumonia caused by SARS-CoV-2 may be associated with rapid virus replication and continual inflammation. Cytokine storm is an abnormal immune activation caused by viruses, which occurs in the third stage or severe stage of the diseases and the stage that can lead to death 44. The storm may interfere with the body's immune system, result in excessive immunization responses, causing diffuse acute lung injury, impairment of ventilation function of lung and a series of critical manifestations 45-47. According to clinical studies, concentrations of diverse associated cytokines were aberrant in COVID-19 patients, such as TNF-α, IL-1β, IL-6, IL-2, and IL-10 3, 48, 49. TNF-α, a typical pro-inflammatory cytokine, the chronically elevated or excessive production of which could trigger a cytokine storm causing cell death during the acute stage of tissue injury 50. IL-1β is also an intense pro-inflammatory cytokine that has received substantial attention 48. Both TNF-α and IL-1β are released from macrophages or monocytes after initial infective or physical insult, may induce the expression of vascular cell adhesion molecule-1 (VCAM*-*1) on endothelial cells to promote adhesion of Neutrophils 51-53. Activation of neutrophils produces reactive oxygen species (ROS), causing lipid peroxidation injury, DNA breakage, protein denaturation, and endothelial cells dysfunction, as well as eventually inducing pulmonary tissue damage 54, 55. Furthermore, cytokines leaking into the bloodstream may contribute to distal organ injury and culminates in multiple organ dysfunction syndromes or even death.

The Ingredient-target network showed that there are 158 ingredients in LHQW-C influencing the 49 genes that may play a role in COVID-19 treatment. As illustrated in PPI network, LHQW-C seemed to widely intervene in the production of inflammatory factors, including IL-6, TNF, IL-1β, and IL-2, especially IL-6, which was suggested to be used as clinical indicators in the prognosis and outcome of COVID-19 patients 46, 49, 56. IL-6 is a cytokine known to be beneficial to host defense against various infections and tissue injuries 57. Nevertheless, excessive IL-6 may cause acute cytokine storm during the anti-infection process, which can further lead to multiple organ dysfunctions and multiple system failures 47, 58. It has been suggested that pathogenic T cells are activated after SARS-CoV-2 infects the human body, which will mediate the production of granulocyte-colony stimulating factor (GM-CSF) and IL-6 59. GM-CSF further activates CD14+ and CD16+ monocytes, generating greater amounts of IL-6 and other inflammatory factors, which could incite cytokine storm and result in severe inflammatory damage to the lungs 60. Therefore, it is an important approach to block the occurrence of cytokine storm and prevent the process of patients with pneumonia by inhibiting the expression of IL-6. As four of the main compounds in LHQW-C, quercetin, luteolin, and wogonin were successfully docked to target IL-6, which suggested that they might limit excessive tissue disruption caused by inflammation cytokine.

Quercetin, a typical flavonoid, is one of the main compounds in LHQW-C has been shown to inhibit TNF-α, IL-6, IL-1β, and IL-2, thereby contributing to anti-inflammatory activity 61-64. Similar to quercetin, kaempferol could suppress TNF-α, IL-1β, and IL-6 65, 66. Other key ingredients also affect the expression of inflammatory cytokine. For example, luteolin inhibits TNF-α, IL-6, IL-1β expression 67-69, wogonin suppresses IL-6 production 70. Clinical evidence has showed that LHQW-C could reduce pro-inflammatory cytokines caused by viruses, such as TNF-α, IL-6 71. Additionally, some experimental data with animal models and *in vitro* models also indicated that LHQW-C has anti-inflammatory properties 72-74, which are consistent with the findings of ours.

Postmortem results and pulmonary pathological collectively indicated that alveolar epithelial cell injury, the hyaline membrane formed in the alveolar cavity, monocyte, and macrophage infiltration. Also, there is severe type 2 alveolar epithelial cell hyperplasia, with some cells exfoliated 75, 76. The alveoli are the main part for gas exchange, as well as the basic units of the lung. Maintaining an intact alveolar-capillary barrier in the lungs is critical to effective gas exchange. The disruption of alveolar endothelial and epithelial barriers causes inflammatory cell infiltration, increased permeability, and extravascular edema fluid accumulation, leading ultimately to alteration in the alveolar-capillary barrier 77-79. Normally, the integrity of the barrier relies on tight junctions (TJs) and adherens junctions (AJs), and basement membrane/extracellular matrix coverage collectively 79, 80. TNF could lead to disturbance in junction localization of zonula occludens-1 (ZO-1) and down-regulation of ZO-1 expression, as well as other claudin family members in the lung, functionally regulating the opening of the TJs barrier 81. As described in Ingredient-target network, the key compounds in LHQW-C could interact with TNF. Meanwhile*,* KEGG enrichment analysis showed that TNF signaling pathway was highly enriched. Through the pathway, LHQW-C might protect alveolar endothelial/epithelial barriers by maintaining the integrity of intercellular junctions, eventually alleviating COVID-19 by improving pulmonary ventilation function.

3CL pro is considered as a promising drug target, which plays a key role in the replication cycle of the virus 82. The active site of which is located in the gap between domains I and II, and has a Cys-His (Cys145 and His41) at the active site 83. Considering that most of the Chinese herbal medicines have direct antiviral activity, it may be an underlying mechanism by which LHQW-C prevents COVID-19 84. Therefore, we also evaluated the interaction between potential active compounds and 3CL pro. Four active compounds and 3CL pro were selected for the second molecular docking. These ingredients were fitted well in the active pocket of 3CL pro, indicating that the 4 compounds may play a key role when LHQW-C suppresses the virus.

Although we have described some interesting findings, there are still some potential limitations in our present study. Firstly, the databases for Chinese herbal medicines are incomplete, their accuracy and integrity cannot be guaranteed. Active compounds obtained from TCMSP with OB ≥30% and DL ≥0.18 may actually haven't good absorption. Additionally, several key compounds obtained by network pharmacology analysis may have false positive defects, but it can be improved using analytical technologies such as ultra-performance liquid chromatography (UPLC), liquid chromatography-tandem mass spectrometry (LC-MS/MS), and high-performance liquid chromatography (HPLC). Secondly, all of the data were based on in-silico analysis, there may be false positive and false negative interactions between compound-protein and protein-protein. Further experiments are needed to validate our findings.

In conclusion, we examined the potential working mechanism of LHQW-C for COVID-19 using an integrating network pharmacology method. Based on clinical 13 and *in vitro* studies 73 as well as our pharmacology network analysis, we can speculate that LHQW-C may have some protective effects in COVID-19 by inhibiting viral replication, suppressing cytokine storm, and protecting the pulmonary alveolar-capillary barrier. Although we have described some encouraging data, further works are needed to validate our bioinformatic and network pharmacology based findings, including HPLC-MS technology based active ingredients analysis, *in vitro* validation of the mechanisms of LHQW-C.

## Supplementary Material

Supplementary tables.

## Figures and Tables

**Table 1:** Molecular docking of four key ingredients from LHQW-C with top 9 therapeutic targets

Compound	Binding Energy/(kcal mol^-1^)								
	IL6	TNF	MAPK1	CASP3	CXCL8	IL10	IL-1B	MAPK8	VEGFA
quercetin	-5.2	-4.7	-8.4	-6.6	-6.8	-7.9	-6.2	-7.2	-4.6
luteolin	-5.7	-5.0	-8.8	-6.6	-6.9	-7.8	-6.4	-7.3	-4.6
wogonin	-5.5	-5.0	-8.3	-5.8	-6.5	-7.0	-6.1	-7.3	-4.5
kaempferol	-5.0	-4.7	-8.1	-6.1	-6.2	-6.8	-6.4	-7.2	-4.7

**Table 2:** Results of molecular docking studies of 4 compounds in the active sites of proteins (3CL pro)

Compound	Structure	Receptor	Binding Energy/(kcal/mol)
quercetin	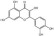	3CL pro	-7.2
luteolin	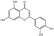	3CL pro	-7.4
wogonin	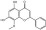	3CL pro	-6.8
kaempferol	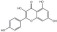	3CL pro	-7.8
