# Supplementary material for: A network pharmacology based approach for predicting active ingredients and potential mechanism of Lianhuaqingwen capsule in treating COVID-19
Source: Int J Med Sci. 2021 Feb 24;18(8):1866–76. doi: 10.7150/ijms.53685 (PMC7976588; doi:10.7150/ijms.53685)
Supplement: Supplementary file 1 — Supplementary tables. [file ijmsv18p1866s1.pdf]

A network pharmacology based approach for predicting active ingredients and potential mechanism of Lianhuaqingwen capsule in treating COVID-19

Xiaobo Zhang[1&], Rui Gao[1&], Zubing Zhou[1], Xuehua Tang[2], Jingjing Lin, Long Wang[1], Xin Zhou[1,*], Tao Shen[1,*]

[1] School of Basic Medicine, Chengdu University of Traditional Chinese Medicine, Chengdu, China.
[2] Academic Department, Zhuhai Ebang Pharmaceutical Co., Ltd. Zhuhai, China.

* Corresponding author

Xin Zhou, School of Basic Medicine, Chengdu University of Traditional Chinese Medicine, Chengdu, 611137 China; Email: cindychouff@163.com.

Tao Shen, School of Basic Medicine, Chengdu University of Traditional Chinese Medicine, Chengdu, 611137 China; Email: shentaotcm@aliyun.com.

& These authors contributed equally to this work.

Supplementary Table 1: 263 ingredients of the 13 herbs of LHQW-C collected from TCMSP, BATMAN, and TCMID.

Herb	Ingredient	Mol ID	OB	DL	Database
Fructus Forsythiae	wogonin	MOL000173	30.68	0.23	TCMSP
Fructus Forsythiae	20(S)-dammar-24-ene-3β,20-diol-3-acetate	MOL003281	40.23	0.82	TCMSP
Fructus Forsythiae	(2R,3R,4S)-4-(4-hydroxy-3-methoxy-phenyl)-7-methoxy-2,3-dimethylol-tetralin-6-ol	MOL003283	66.51	0.39	TCMSP
Fructus Forsythiae	(3R,4R)-3,4-bis[(3,4-dimethoxyphenyl)methyl]oxolan-2-one	MOL003290	52.3	0.48	TCMSP
Fructus Forsythiae	(+)-pinoresinol monomethyl ether	MOL003295	53.08	0.57	TCMSP
Fructus Forsythiae	PHILLYRIN	MOL003305	36.4	0.86	TCMSP
Fructus Forsythiae	ACon1_001697	MOL003306	85.12	0.57	TCMSP
Fructus Forsythiae	(+)-pinoresinol monomethyl ether-4-D-beta-glucoside_qt	MOL003308	61.2	0.57	TCMSP
Fructus Forsythiae	3beta-Acetyl-20,25-epoxydammarane-24alpha-ol	MOL003315	33.07	0.79	TCMSP
Fructus Forsythiae	Mairin	MOL000211	55.38	0.78	TCMSP
Fructus Forsythiae	FORSYTHINOL	MOL003322	81.25	0.57	TCMSP
Fructus Forsythiae	(-)-Phillygenin	MOL003330	95.04	0.57	TCMSP
Fructus Forsythiae	β-amyrin acetate	MOL003344	42.06	0.74	TCMSP
Fructus Forsythiae	hyperforin	MOL003347	44.03	0.6	TCMSP
Fructus Forsythiae	adhyperforin	MOL003348	44.03	0.61	TCMSP
Fructus Forsythiae	Lactucasterol	MOL003365	40.99	0.85	TCMSP
Fructus Forsythiae	Onjixanthone I	MOL003370	79.16	0.3	TCMSP
Fructus Forsythiae	beta-sitosterol	MOL000358	36.91	0.75	TCMSP
Fructus Forsythiae	kaempferol	MOL000422	41.88	0.24	TCMSP
Fructus Forsythiae	arctiin	MOL000522	34.45	0.84	TCMSP
Fructus Forsythiae	luteolin	MOL000006	36.16	0.25	TCMSP
Fructus Forsythiae	bicuculline	MOL000791	69.67	0.88	TCMSP
Fructus Forsythiae	quercetin	MOL000098	46.43	0.28	TCMSP
Flos Lonicerae Japonicae	Mandenol	MOL001494	42	0.19	TCMSP
Flos Lonicerae Japonicae	Ethyl linolenate	MOL001495	46.1	0.2	TCMSP
Flos Lonicerae Japonicae	phytofluene	MOL002707	43.18	0.5	TCMSP
Flos Lonicerae Japonicae	Eriodyctiol (flavanone)	MOL002914	41.35	0.24	TCMSP
Flos Lonicerae Japonicae	(-)-(3R,8S,9R,9aS,10aS)-9-ethenyl-8-(beta-D-glucopyranosyloxy)-2,3,9,9a,10,10a-hexahydro-5-oxo-	MOL003006	87.47	0.23	TCMSP

	5H,8H-pyrano[4,3-d]oxazolo[3,2-a]pyridine-3-carboxylic acid_qt				
Flos Lonicerae Japonicae	secologanic dibutylacetal_qt	MOL003014	53.65	0.29	TCMSP
Flos Lonicerae Japonicae	beta-carotene	MOL002773	37.18	0.58	TCMSP
Flos Lonicerae Japonicae	ZINC03978781	MOL003036	43.83	0.76	TCMSP
Flos Lonicerae Japonicae	Chryseriol	MOL003044	35.85	0.27	TCMSP
Flos Lonicerae Japonicae	kryptoxanthin	MOL003059	47.25	0.57	TCMSP
Flos Lonicerae Japonicae	4,5'-Retro-.beta.,.beta.-Carotene-3,3'-dione, 4',5'-didehydro-	MOL003062	31.22	0.55	TCMSP
Flos Lonicerae Japonicae	5-hydroxy-7-methoxy-2-(3,4,5-trimethoxyphenyl)chromone	MOL003095	51.96	0.41	TCMSP
Flos Lonicerae Japonicae	7-epi-Vogeloside	MOL003101	46.13	0.58	TCMSP
Flos Lonicerae Japonicae	Caeruloside C	MOL003108	55.64	0.73	TCMSP
Flos Lonicerae Japonicae	Centauroside_qt	MOL003111	55.79	0.5	TCMSP
Flos Lonicerae Japonicae	Ioniceracetalides B_qt	MOL003117	61.19	0.19	TCMSP
Flos Lonicerae Japonicae	XYLOSTOSIDINE	MOL003124	43.17	0.64	TCMSP
Flos Lonicerae Japonicae	dinethylsecologanoside	MOL003128	48.46	0.48	TCMSP
Flos Lonicerae Japonicae	beta-sitosterol	MOL000358	36.91	0.75	TCMSP
Flos Lonicerae Japonicae	kaempferol	MOL000422	41.88	0.24	TCMSP
Flos Lonicerae Japonicae	Stigmasterol	MOL000449	43.83	0.76	TCMSP
Flos Lonicerae Japonicae	luteolin	MOL000006	36.16	0.25	TCMSP
Flos Lonicerae Japonicae	quercetin	MOL000098	46.43	0.28	TCMSP
Herba Ephedrae	leucopelargonidin	MOL010788	57.97	0.24	TCMSP
Herba Ephedrae	Herbacetin	MOL002823	36.07	0.27	TCMSP
Herba Ephedrae	Resivit	MOL010489	30.84	0.27	TCMSP
Herba Ephedrae	kaempferol	MOL000422	41.88	0.24	TCMSP
Herba Ephedrae	delphinidin	MOL004798	40.63	0.28	TCMSP
Herba Ephedrae	quercetin	MOL000098	46.43	0.28	TCMSP

Herba Ephedrae	luteolin	MOL000006	36.16	0.25	TCMSP
Herba Ephedrae	beta-sitosterol	MOL000358	36.91	0.75	TCMSP
Herba Ephedrae	Stigmasterol	MOL000449	43.83	0.76	TCMSP
Herba Ephedrae	(+)-catechin	MOL000492	54.83	0.24	TCMSP
Herba Ephedrae	Mandenol	MOL001494	42	0.19	TCMSP
Herba Ephedrae	Supraene	MOL001506	33.55	0.42	TCMSP
Herba Ephedrae	24-Ethylcholest-4-en-3-one	MOL001755	36.08	0.76	TCMSP
Herba Ephedrae	poriferast-5-en-3beta-ol	MOL001771	36.91	0.75	TCMSP
Herba Ephedrae	Diosmetin	MOL002881	31.14	0.27	TCMSP
Herba Ephedrae	naringenin	MOL004328	59.29	0.21	TCMSP
Herba Ephedrae	taxifolin	MOL004576	57.84	0.27	TCMSP
Herba Ephedrae	campest-5-en-3beta-ol	MOL005043	37.58	0.71	TCMSP
Herba Ephedrae	eriodictyol	MOL005190	71.79	0.24	TCMSP
Herba Ephedrae	Genkwanin	MOL005573	37.13	0.24	TCMSP
Herba Ephedrae	Pectolinarigenin	MOL005842	41.17	0.3	TCMSP
Herba Ephedrae	(+)-Leucocyanidin	MOL007214	37.61	0.27	TCMSP
Herba Ephedrae	Truflex OBP	MOL011319	43.74	0.24	TCMSP
Almond	estrone	MOL010921	53.56	0.32	TCMSP
Almond	Diisooctyl succinate	MOL010922	31.62	0.23	TCMSP
Almond	11,14-eicosadienoic acid	MOL002211	39.99	0.2	TCMSP
Almond	(6Z,10E,14E,18E)-2,6,10,15,19,23-hexamethyltetracosa-2,6,10,14,18,22-hexaene	MOL002372	33.55	0.42	TCMSP
Almond	sitosterol	MOL000359	36.91	0.75	TCMSP
Almond	Stigmasterol	MOL000449	43.83	0.76	TCMSP
Almond	gondoic acid	MOL005030	30.7	0.2	TCMSP
Almond	CLR	MOL000953	37.87	0.68	TCMSP
Almond	Mairin	MOL000211	55.38	0.78	TCMSP
Almond	(+)-catechin	MOL000492	54.83	0.24	TCMSP
Almond	Glycyrol	MOL002311	90.78	0.67	TCMSP
Almond	Ziziphin_qt	MOL003410	66.95	0.62	TCMSP
Almond	Spinasterol	MOL004355	42.98	0.76	TCMSP
Almond	Licochalcone B	MOL004841	76.76	0.19	TCMSP
Almond	liquiritin	MOL004903	65.69	0.74	TCMSP
Almond	Glabridin	MOL004908	53.25	0.47	TCMSP
Almond	Phaseol	MOL005017	78.77	0.58	TCMSP
Almond	Machiline	MOL007207	79.64	0.24	TCMSP
Almond	l-SPD	MOL012922	87.35	0.54	TCMSP
Radix Isatidis	acacetin	MOL001689	34.97	0.24	TCMSP
Radix Isatidis	isovitexin	MOL002322	31.29	0.72	TCMSP
Radix Isatidis	Isaindigodione	MOL001721	60.12	0.41	TCMSP
Radix Isatidis	2-O-beta-D-glucopyranosyl-2H-1,4-benzoxazin-3(4H)-one	MOL001722	43.62	0.31	TCMSP

Radix Isatidis	pinoresinol-4-O-beta-D-apiosyl-beta-D-glucopyranoside	MOL001726	36.45	0.51	TCMSP
Radix Isatidis	3-[2′ -(5′ - hydroxymethyl) furyl] -1 (2H) -isoquinolinone-7-O-BETA-D-glucoside_qt	MOL001728	51.74	0.18	TCMSP
Radix Isatidis	EUPATORIN	MOL001733	30.23	0.37	TCMSP
Radix Isatidis	3-[[(2R,3R,5R,6S)-3,5-dihydroxy-6-(1H-indol-3-yloxy)-4-oxooxan-2-yl]methoxy]-3-oxopropanoic acid	MOL001734	85.87	0.47	TCMSP
Radix Isatidis	Dinatin	MOL001735	30.97	0.27	TCMSP
Radix Isatidis	(-)-taxifolin	MOL001736	60.51	0.27	TCMSP
Radix Isatidis	ZINC03860434	MOL001749	43.59	0.35	TCMSP
Radix Isatidis	glucobrassicin	MOL001750	66.02	0.48	TCMSP
Radix Isatidis	24-Ethylcholest-4-en-3-one	MOL001755	36.08	0.76	TCMSP
Radix Isatidis	quindoline	MOL001756	33.17	0.22	TCMSP
Radix Isatidis	hydroxyindirubin	MOL001767	63.37	0.3	TCMSP
Radix Isatidis	beta-sitosterol dodecantate	MOL001769	34.57	0.57	TCMSP
Radix Isatidis	poriferast-5-en-3beta-ol	MOL001771	36.91	0.75	TCMSP
Radix Isatidis	Ineketone	MOL001774	37.14	0.3	TCMSP
Radix Isatidis	Sinoacutine	MOL001779	49.11	0.46	TCMSP
Radix Isatidis	Indigo	MOL001781	38.2	0.26	TCMSP
Radix Isatidis	(2Z)-2-(2-oxoindolin-3-ylidene)indolin-3-one	MOL001782	48.4	0.26	TCMSP
Radix Isatidis	2-(9-((3-methyl-2-oxopent-3-en-1-yl)oxy)-2-oxo-1,2,8,9-tetrahydrofuro[2,3-h]quinolin-8-yl)propan-2-yl acetate	MOL001783	64	0.57	TCMSP
Radix Isatidis	Linarin	MOL001790	39.84	0.71	TCMSP
Radix Isatidis	DFV	MOL001792	32.76	0.18	TCMSP
Radix Isatidis	(E)-2-[(3-indole)cyanomethylene-]-3-indolinone	MOL001793	54.59	0.32	TCMSP
Radix Isatidis	neohesperidin_qt	MOL001798	71.17	0.27	TCMSP
Radix Isatidis	rosasterol	MOL001800	35.87	0.75	TCMSP
Radix Isatidis	Sinensetin	MOL001803	50.56	0.45	TCMSP
Radix Isatidis	Stigmasta-5,22-diene-3beta,7alpha-diol	MOL001804	43.04	0.82	TCMSP
Radix Isatidis	Stigmasta-5,22-diene-3beta,7beta-diol	MOL001806	42.56	0.83	TCMSP
Radix Isatidis	6-(3-oxoindolin-2-ylidene)indolo[2,1-b]quinazolin-12-one	MOL001810	45.28	0.89	TCMSP
Radix Isatidis	(E)-3-(3,5-dimethoxy-4-hydroxy-benzylidene)-2-indolinone	MOL001814	57.18	0.25	TCMSP
Radix Isatidis	(E)-3-(3,5-dimethoxy-4-hydroxyb-enzylidene)-2-indolinone	MOL001820	65.17	0.25	TCMSP

Radix Isatidis	3-[(3,5-dimethoxy-4-oxo-1-cyclohexa-2,5-dienylidene)methyl]-2,4-dihydro-1H-pyrrolo[2,1-b]quinazolin-9-one	MOL001828	51.84	0.56	TCMSP
Radix Isatidis	Glucobrassicin-1-Sulfonate_qt	MOL001833	42.52	0.24	TCMSP
Radix Isatidis	beta-sitosterol	MOL000358	36.91	0.75	TCMSP
Radix Isatidis	sitosterol	MOL000359	36.91	0.75	TCMSP
Radix Isatidis	Stigmasterol	MOL000449	43.83	0.76	TCMSP
Radix Isatidis	CLR	MOL000953	37.87	0.68	TCMSP
Fortunes Boss fern Rhizome	(2R)-5,7-dihydroxy-2-(4-hydroxyphenyl)chroman-4-one	MOL001040	42.36	0.21	TCMSP
Fortunes Boss fern Rhizome	11-Hydroxynumantenine	MOL002605	50.79	0.71	TCMSP
Fortunes Boss fern Rhizome	Harmonyl	MOL002609	43.8	0.46	TCMSP
Fortunes Boss fern Rhizome	ZINC00035529	MOL002610	58.39	0.22	TCMSP
Fortunes Boss fern Rhizome	Flavidin	MOL002614	30.1	0.26	TCMSP
Fortunes Boss fern Rhizome	Albaspidin AA	MOL002619	31.16	0.36	TCMSP
Fortunes Boss fern Rhizome	kaempferol	MOL000422	41.88	0.24	TCMSP
Herba Houttuyniae	Isoramanone	MOL003851	39.97	0.51	TCMSP
Herba Houttuyniae	kaempferol	MOL000422	41.88	0.24	TCMSP
Herba Houttuyniae	1-methyl-2-nonacosyl-4-quinolone	MOL004345	31.54	0.5	TCMSP
Herba Houttuyniae	Ruvoside_qt	MOL004350	36.12	0.76	TCMSP
Herba Houttuyniae	C09747	MOL004351	37.28	0.25	TCMSP
Herba Houttuyniae	Spinasterol	MOL004355	42.98	0.76	TCMSP
Herba Houttuyniae	quercetin	MOL000098	46.43	0.28	TCMSP
Herba Pogostemonis	Diop	MOL002879	43.59	0.39	TCMSP
Herba Pogostemonis	Genkwanin	MOL005573	37.13	0.24	TCMSP
Herba Pogostemonis	patchoulan 1,12-diol	MOL005884	38.17	0.25	TCMSP
Herba Pogostemonis	pachypodol	MOL005890	75.06	0.4	TCMSP
Herba Pogostemonis	5-Hydroxy-7,4'-dimethoxyflavanon	MOL005911	51.54	0.27	TCMSP
Herba Pogostemonis	irisolidone	MOL005916	37.78	0.3	TCMSP
Herba Pogostemonis	phenanthrone	MOL005918	38.7	0.33	TCMSP
Herba Pogostemonis	quercetin 7-O-β-D-glucoside	MOL005921	49.57	0.27	TCMSP
Herba Pogostemonis	Acanthoside B	MOL005922	43.35	0.77	TCMSP
Herba Pogostemonis	3,23-dihydroxy-12-oleanen-28-oic acid	MOL005923	30.86	0.86	TCMSP
Herba Pogostemonis	quercetin	MOL000098	46.43	0.28	TCMSP
Rheum palmatum	EUPATIN	MOL002235	50.8	0.41	TCMSP
Rheum palmatum	Mutatochrome	MOL002251	48.64	0.61	TCMSP
Rheum palmatum	Physciondiglucoside	MOL002259	41.65	0.63	TCMSP
Rheum palmatum	Procyanidin B-5,3'-O-gallate	MOL002260	31.99	0.32	TCMSP

Rheum palmatum	rhein	MOL002268	47.07	0.28	TCMSP
Rheum palmatum	Sennoside E_qt	MOL002276	50.69	0.61	TCMSP
Rheum palmatum	Torachrysone-8-O-beta-D-(6'-oxayl)-glucoside	MOL002280	43.02	0.74	TCMSP
Rheum palmatum	Toralactone	MOL002281	46.46	0.24	TCMSP
Rheum palmatum	Emodin-1-O-beta-D-glucopyranoside	MOL002288	44.81	0.8	TCMSP
Rheum palmatum	Sennoside D_qt	MOL002293	61.06	0.61	TCMSP
Rheum palmatum	Daucosterol_qt	MOL002297	35.89	0.7	TCMSP
Rheum palmatum	palmidin A	MOL002303	32.45	0.65	TCMSP
Rheum palmatum	beta-sitosterol	MOL000358	36.91	0.75	TCMSP
Rheum palmatum	aloe-emodin	MOL000471	83.38	0.24	TCMSP
Rheum palmatum	gallic acid-3-O-(6'-O-galloyl)-glucoside	MOL000554	30.25	0.67	TCMSP
Rheum palmatum	(-)-catechin	MOL000096	49.68	0.24	TCMSP
Glycyrrhiza uralensis Fisch	Inermine	MOL001484	75.18	0.54	TCMSP
Glycyrrhiza uralensis Fisch	DFV	MOL001792	32.76	0.18	TCMSP
Glycyrrhiza uralensis Fisch	Mairin	MOL000211	55.38	0.78	TCMSP
Glycyrrhiza uralensis Fisch	Glycyrol	MOL002311	90.78	0.67	TCMSP
Glycyrrhiza uralensis Fisch	Jaranol	MOL000239	50.83	0.29	TCMSP
Glycyrrhiza uralensis Fisch	Medicarpin	MOL002565	49.22	0.34	TCMSP
Glycyrrhiza uralensis Fisch	isorhamnetin	MOL000354	49.6	0.31	TCMSP
Glycyrrhiza uralensis Fisch	sitosterol	MOL000359	36.91	0.75	TCMSP
Glycyrrhiza uralensis Fisch	Lupiwighteone	MOL003656	51.64	0.37	TCMSP
Glycyrrhiza uralensis Fisch	7-Methoxy-2-methyl isoflavone	MOL003896	42.56	0.2	TCMSP
Glycyrrhiza uralensis Fisch	formononetin	MOL000392	69.67	0.21	TCMSP
Glycyrrhiza uralensis Fisch	Calycosin	MOL000417	47.75	0.24	TCMSP
Glycyrrhiza uralensis Fisch	kaempferol	MOL000422	41.88	0.24	TCMSP
Glycyrrhiza uralensis Fisch	naringenin	MOL004328	59.29	0.21	TCMSP
Glycyrrhiza uralensis Fisch	(2S)-2-[4-hydroxy-3-(3-methylbut-2-enyl)phenyl]-8,8-dimethyl-2,3-dihydropyrano[2,3-f]chromen-4-one	MOL004805	31.79	0.72	TCMSP

Glycyrrhiza uralensis Fisch	euchrenone	MOL004806	30.29	0.57	TCMSP
Glycyrrhiza uralensis Fisch	glyasperin B	MOL004808	65.22	0.44	TCMSP
Glycyrrhiza uralensis Fisch	glyasperin F	MOL004810	75.84	0.54	TCMSP
Glycyrrhiza uralensis Fisch	Glyasperin C	MOL004811	45.56	0.4	TCMSP
Glycyrrhiza uralensis Fisch	Isotrifoliol	MOL004814	31.94	0.42	TCMSP
Glycyrrhiza uralensis Fisch	(E)-1-(2,4-dihydroxyphenyl)-3-(2,2-dimethylchromen-6-yl)prop-2-en-1-one	MOL004815	39.62	0.35	TCMSP
Glycyrrhiza uralensis Fisch	kanzonols W	MOL004820	50.48	0.52	TCMSP
Glycyrrhiza uralensis Fisch	(2S)-6-(2,4-dihydroxyphenyl)-2-(2-hydroxypropan-2-yl)-4-methoxy-2,3-dihydrofuro[3,2-g]chromen-7-one	MOL004824	60.25	0.63	TCMSP
Glycyrrhiza uralensis Fisch	Semilicoisoflavone B	MOL004827	48.78	0.55	TCMSP
Glycyrrhiza uralensis Fisch	Glepidotin A	MOL004828	44.72	0.35	TCMSP
Glycyrrhiza uralensis Fisch	Glepidotin B	MOL004829	64.46	0.34	TCMSP
Glycyrrhiza uralensis Fisch	Phaseolinisoflavan	MOL004833	32.01	0.45	TCMSP
Glycyrrhiza uralensis Fisch	Glypallichalcone	MOL004835	61.6	0.19	TCMSP
Glycyrrhiza uralensis Fisch	8-(6-hydroxy-2-benzofuranyl)-2,2-dimethyl-5-chromenol	MOL004838	58.44	0.38	TCMSP
Glycyrrhiza uralensis Fisch	Licochalcone B	MOL004841	76.76	0.19	TCMSP
Glycyrrhiza uralensis Fisch	licochalcone G	MOL004848	49.25	0.32	TCMSP
Glycyrrhiza uralensis Fisch	3-(2,4-dihydroxyphenyl)-8-(1,1-dimethylprop-2-enyl)-7-hydroxy-5-methoxy-coumarin	MOL004849	59.62	0.43	TCMSP
Glycyrrhiza uralensis Fisch	Licoricone	MOL004855	63.58	0.47	TCMSP
Glycyrrhiza uralensis Fisch	Gancaonin A	MOL004856	51.08	0.4	TCMSP
Glycyrrhiza uralensis Fisch	Gancaonin B	MOL004857	48.79	0.45	TCMSP
Glycyrrhiza uralensis Fisch	licorice glycoside E	MOL004860	32.89	0.27	TCMSP

Glycyrrhiza uralensis Fisch	3-(3,4-dihydroxyphenyl)-5,7-dihydroxy-8-(3-methylbut-2-enyl)chromone	MOL004863	66.37	0.41	TCMSP
Glycyrrhiza uralensis Fisch	5,7-dihydroxy-3-(4-methoxyphenyl)-8-(3-methylbut-2-enyl)chromone	MOL004864	30.49	0.41	TCMSP
Glycyrrhiza uralensis Fisch	2-(3,4-dihydroxyphenyl)-5,7-dihydroxy-6-(3-methylbut-2-enyl)chromone	MOL004866	44.15	0.41	TCMSP
Glycyrrhiza uralensis Fisch	Glycyrin	MOL004879	52.61	0.47	TCMSP
Glycyrrhiza uralensis Fisch	Licocoumarone	MOL004882	33.21	0.36	TCMSP
Glycyrrhiza uralensis Fisch	Licoisoflavone	MOL004883	41.61	0.42	TCMSP
Glycyrrhiza uralensis Fisch	Licoisoflavone B	MOL004884	38.93	0.55	TCMSP
Glycyrrhiza uralensis Fisch	licoisoflavanone	MOL004885	52.47	0.54	TCMSP
Glycyrrhiza uralensis Fisch	shinpterocarpin	MOL004891	80.3	0.73	TCMSP
Glycyrrhiza uralensis Fisch	(E)-3-[3,4-dihydroxy-5-(3-methylbut-2-enyl)phenyl]-1-(2,4-dihydroxyphenyl)prop-2-en-1-one	MOL004898	46.27	0.31	TCMSP
Glycyrrhiza uralensis Fisch	liquiritin	MOL004903	65.69	0.74	TCMSP
Glycyrrhiza uralensis Fisch	licopyranocoumarin	MOL004904	80.36	0.65	TCMSP
Glycyrrhiza uralensis Fisch	3,22-Dihydroxy-11-oxo-delta(12)-oleanene-27-alpha-methoxycarbonyl-29-oic acid	MOL004905	34.32	0.55	TCMSP
Glycyrrhiza uralensis Fisch	Glyzaglabrin	MOL004907	61.07	0.35	TCMSP
Glycyrrhiza uralensis Fisch	Glabridin	MOL004908	53.25	0.47	TCMSP
Glycyrrhiza uralensis Fisch	Glabranin	MOL004910	52.9	0.31	TCMSP
Glycyrrhiza uralensis Fisch	Glabrene	MOL004911	46.27	0.44	TCMSP
Glycyrrhiza uralensis Fisch	Glabrone	MOL004912	52.51	0.5	TCMSP
Glycyrrhiza uralensis Fisch	1,3-dihydroxy-9-methoxy-6-benzofurano[3,2-c]chromenone	MOL004913	48.14	0.43	TCMSP
Glycyrrhiza uralensis Fisch	1,3-dihydroxy-8,9-dimethoxy-6-benzofurano[3,2-c]chromenone	MOL004914	62.9	0.53	TCMSP

Glycyrrhiza uralensis Fisch	Eurycarpin A	MOL004915	43.28	0.37	TCMSP
Glycyrrhiza uralensis Fisch	glycyroside	MOL004917	37.25	0.79	TCMSP
Glycyrrhiza uralensis Fisch	(-)-Medicocarpin	MOL004924	40.99	0.95	TCMSP
Glycyrrhiza uralensis Fisch	Sigmoidin-B	MOL004935	34.88	0.41	TCMSP
Glycyrrhiza uralensis Fisch	(2R)-7-hydroxy-2-(4-hydroxyphenyl)chroman-4-one	MOL004941	71.12	0.18	TCMSP
Glycyrrhiza uralensis Fisch	(2S)-7-hydroxy-2-(4-hydroxyphenyl)-8-(3-methylbut-2-enyl)chroman-4-one	MOL004945	36.57	0.32	TCMSP
Glycyrrhiza uralensis Fisch	Isoglycyrol	MOL004948	44.7	0.84	TCMSP
Glycyrrhiza uralensis Fisch	Isolicoflavonol	MOL004949	45.17	0.42	TCMSP
Glycyrrhiza uralensis Fisch	HMO	MOL004957	38.37	0.21	TCMSP
Glycyrrhiza uralensis Fisch	1-Methoxyphaseollidin	MOL004959	69.98	0.64	TCMSP
Glycyrrhiza uralensis Fisch	Quercetin der.	MOL004961	46.45	0.33	TCMSP
Glycyrrhiza uralensis Fisch	3'-Hydroxy-4'-O-Methylglabridin	MOL004966	43.71	0.57	TCMSP
Glycyrrhiza uralensis Fisch	licochalcone a	MOL000497	40.79	0.29	TCMSP
Glycyrrhiza uralensis Fisch	3'-Methoxyglabridin	MOL004974	46.16	0.57	TCMSP
Glycyrrhiza uralensis Fisch	2-[(3R)-8,8-dimethyl-3,4-dihydro-2H-pyrano[6,5-f]chromen-3-yl]-5-methoxyphenol	MOL004978	36.21	0.52	TCMSP
Glycyrrhiza uralensis Fisch	Inflacoumarin A	MOL004980	39.71	0.33	TCMSP
Glycyrrhiza uralensis Fisch	icos-5-enoic acid	MOL004985	30.7	0.2	TCMSP
Glycyrrhiza uralensis Fisch	Kanzonol F	MOL004988	32.47	0.89	TCMSP
Glycyrrhiza uralensis Fisch	6-prenylated eriodictyol	MOL004989	39.22	0.41	TCMSP
Glycyrrhiza uralensis Fisch	7,2',4'-trihydroxy－5-methoxy-3－arylcoumarin	MOL004990	83.71	0.27	TCMSP
Glycyrrhiza uralensis Fisch	7-Acetoxy-2-methylisoflavone	MOL004991	38.92	0.26	TCMSP

Glycyrrhiza uralensis Fisch	8-prenylated eriodictyol	MOL004993	53.79	0.4	TCMSP
Glycyrrhiza uralensis Fisch	gadelaidic acid	MOL004996	30.7	0.2	TCMSP
Glycyrrhiza uralensis Fisch	Vestitol	MOL000500	74.66	0.21	TCMSP
Glycyrrhiza uralensis Fisch	Gancaonin G	MOL005000	60.44	0.39	TCMSP
Glycyrrhiza uralensis Fisch	Gancaonin H	MOL005001	50.1	0.78	TCMSP
Glycyrrhiza uralensis Fisch	Licoagrocarpin	MOL005003	58.81	0.58	TCMSP
Glycyrrhiza uralensis Fisch	Glyasperins M	MOL005007	72.67	0.59	TCMSP
Glycyrrhiza uralensis Fisch	Glycyrrhiza flavonol A	MOL005008	41.28	0.6	TCMSP
Glycyrrhiza uralensis Fisch	Licoagroisoflavone	MOL005012	57.28	0.49	TCMSP
Glycyrrhiza uralensis Fisch	18α-hydroxyglycyrrhetic acid	MOL005013	41.16	0.71	TCMSP
Glycyrrhiza uralensis Fisch	Odoratin	MOL005016	49.95	0.3	TCMSP
Glycyrrhiza uralensis Fisch	Phaseol	MOL005017	78.77	0.58	TCMSP
Glycyrrhiza uralensis Fisch	Xambioona	MOL005018	54.85	0.87	TCMSP
Glycyrrhiza uralensis Fisch	dehydroglyasperins C	MOL005020	53.82	0.37	TCMSP
Glycyrrhiza uralensis Fisch	quercetin	MOL000098	46.43	0.28	TCMSP
Gypsum Fibrosum	caso4·2h2o	—	—	—	TCMID
Mentha haplocalyx Briq	Menthol	—	—	—	Batman
Rhodiola rosea	salidroside	—	—	—	Batman

Supplementary Table 2: 226 ingredients in LHQW-C collected from the public databases.

Ingredient	OB	DL	Database
wogonin	30.68	0.23	TCMSP
20(S)-dammar-24-ene-3β,20-diol-3-acetate	40.23	0.82	TCMSP
(2R,3R,4S)-4-(4-hydroxy-3-methoxy-phenyl)-7-methoxy-2,3-dimethylol-tetralin-6-ol	66.51	0.39	TCMSP
(3R,4R)-3,4-bis[(3,4-dimethoxyphenyl)methyl]oxolan-2-one	52.3	0.48	TCMSP
(+)-pinoresinol monomethyl ether	53.08	0.57	TCMSP
PHILLYRIN	36.4	0.86	TCMSP
ACon1_001697	85.12	0.57	TCMSP
(+)-pinoresinol monomethyl ether-4-D-beta-glucoside_qt	61.2	0.57	TCMSP
3beta-Acetyl-20,25-epoxydammarane-24alpha-ol	33.07	0.79	TCMSP
Mairin	55.38	0.78	TCMSP
FORSYTHINOL	81.25	0.57	TCMSP
(-)-Phillygenin	95.04	0.57	TCMSP
β-amyrin acetate	42.06	0.74	TCMSP
hyperforin	44.03	0.6	TCMSP
adhyperforin	44.03	0.61	TCMSP
Lactucasterol	40.99	0.85	TCMSP
Onjixanthone I	79.16	0.3	TCMSP
beta-sitosterol	36.91	0.75	TCMSP
kaempferol	41.88	0.24	TCMSP
arctiin	34.45	0.84	TCMSP
luteolin	36.16	0.25	TCMSP
bicuculline	69.67	0.88	TCMSP
quercetin	46.43	0.28	TCMSP
Mandenol	42	0.19	TCMSP
Ethyl linolenate	46.1	0.2	TCMSP
phytofluene	43.18	0.5	TCMSP
Eriodyctiol (flavanone)	41.35	0.24	TCMSP
(-)-(3R,8S,9R,9aS,10aS)-9-ethenyl-8-(beta-D-glucopyranosyloxy)-2,3,9,9a,10,10a-hexahydro-5-oxo-5H,8H-pyrano[4,3-d]oxazolo[3,2-a]pyridine-3-carboxylic acid_qt	87.47	0.23	TCMSP
secologanic dibutylacetal_qt	53.65	0.29	TCMSP
beta-carotene	37.18	0.58	TCMSP
ZINC03978781	43.83	0.76	TCMSP
Chryseriol	35.85	0.27	TCMSP
kryptoxanthin	47.25	0.57	TCMSP
4,5'-Retro-.beta.,.beta.-Carotene-3,3'-dione, 4',5'-didehydro-	31.22	0.55	TCMSP
5-hydroxy-7-methoxy-2-(3,4,5-trimethoxyphenyl)chromone	51.96	0.41	TCMSP

7-epi-Vogeloside	46.13	0.58	TCMSP
Caeruloside C	55.64	0.73	TCMSP
Centauroside_qt	55.79	0.5	TCMSP
Ioniceracetalides B_qt	61.19	0.19	TCMSP
XYLOSTOSIDINE	43.17	0.64	TCMSP
dinethylsecologanoside	48.46	0.48	TCMSP
Stigmasterol	43.83	0.76	TCMSP
leucopelargonidin	57.97	0.24	TCMSP
Herbacetin	36.07	0.27	TCMSP
Resivit	30.84	0.27	TCMSP
delphinidin	40.63	0.28	TCMSP
(+)-catechin	54.83	0.24	TCMSP
Supraene	33.55	0.42	TCMSP
24-Ethylcholest-4-en-3-one	36.08	0.76	TCMSP
poriferast-5-en-3beta-ol	36.91	0.75	TCMSP
Diosmetin	31.14	0.27	TCMSP
naringenin	59.29	0.21	TCMSP
taxifolin	57.84	0.27	TCMSP
campest-5-en-3beta-ol	37.58	0.71	TCMSP
eriodictyol	71.79	0.24	TCMSP
Genkwanin	37.13	0.24	TCMSP
Pectolinarigenin	41.17	0.3	TCMSP
(+)-Leucocyanidin	37.61	0.27	TCMSP
Truflex OBP	43.74	0.24	TCMSP
estrone	53.56	0.32	TCMSP
Diisooctyl succinate	31.62	0.23	TCMSP
11,14-eicosadienoic acid	39.99	0.2	TCMSP
(6Z,10E,14E,18E)-2,6,10,15,19,23-hexamethyltetracosa-2,6,10,14,18,22-hexaene	33.55	0.42	TCMSP
sitosterol	36.91	0.75	TCMSP
gondoic acid	30.7	0.2	TCMSP
CLR	37.87	0.68	TCMSP
Glycyrol	90.78	0.67	TCMSP
Ziziphin_qt	66.95	0.62	TCMSP
Spinasterol	42.98	0.76	TCMSP
Licochalcone B	76.76	0.19	TCMSP
liquiritin	65.69	0.74	TCMSP
Glabridin	53.25	0.47	TCMSP
Phaseol	78.77	0.58	TCMSP
Machiline	79.64	0.24	TCMSP
l-SPD	87.35	0.54	TCMSP

acacetin	34.97	0.24	TCMSP
isovitexin	31.29	0.72	TCMSP
Isaindigodione	60.12	0.41	TCMSP
2-O-beta-D-glucopyranosyl-2H-1,4-benzoxazin-3(4H)-one	43.62	0.31	TCMSP
pinoresinol-4-O-beta-D-apiosyl-beta-D-glucopyranoside	36.45	0.51	TCMSP
3-[2′ -(5′ - hydroxymethyl) furyl] -1 (2H) -isoquinolinone-7-O-BETA-D-glucoside_qt	51.74	0.18	TCMSP
EUPATORIN	30.23	0.37	TCMSP
3-[[(2R,3R,5R,6S)-3,5-dihydroxy-6-(1H-indol-3-yloxy)-4-oxooxan-2-yl]methoxy]-3-oxopropanoic acid	85.87	0.47	TCMSP
Dinatin	30.97	0.27	TCMSP
(-)-taxifolin	60.51	0.27	TCMSP
ZINC03860434	43.59	0.35	TCMSP
glucobrassicin	66.02	0.48	TCMSP
quindoline	33.17	0.22	TCMSP
hydroxyindirubin	63.37	0.3	TCMSP
beta-sitosterol dodecantate	34.57	0.57	TCMSP
Ineketone	37.14	0.3	TCMSP
Sinoacutine	49.11	0.46	TCMSP
Indigo	38.2	0.26	TCMSP
(2Z)-2-(2-oxoindolin-3-ylidene)indolin-3-one	48.4	0.26	TCMSP
2-(9-((3-methyl-2-oxopent-3-en-1-yl)oxy)-2-oxo-1,2,8,9-tetrahydrofuro[2,3-h]quinolin-8-yl)propan-2-yl acetate	64	0.57	TCMSP
Linarin	39.84	0.71	TCMSP
DFV	32.76	0.18	TCMSP
(E)-2-[(3-indole)cyanomethylene-]-3-indolinone	54.59	0.32	TCMSP
neohesperidin_qt	71.17	0.27	TCMSP
rosasterol	35.87	0.75	TCMSP
Sinensetin	50.56	0.45	TCMSP
Stigmasta-5,22-diene-3beta,7alpha-diol	43.04	0.82	TCMSP
Stigmasta-5,22-diene-3beta,7beta-diol	42.56	0.83	TCMSP
6-(3-oxoindolin-2-ylidene)indolo[2,1-b]quinazolin-12-one	45.28	0.89	TCMSP
(E)-3-(3,5-dimethoxy-4-hydroxy-benzylidene)-2-indolinone	57.18	0.25	TCMSP
(E)-3-(3,5-dimethoxy-4-hydroxyb-enzylidene)-2-indolinone	65.17	0.25	TCMSP
3-[(3,5-dimethoxy-4-oxo-1-cyclohexa-2,5-dienylidene)methyl]-2,4-dihydro-1H-pyrrolo[2,1-b]quinazolin-9-one	51.84	0.56	TCMSP
Glucobrassicin-1-Sulfonate_qt	42.52	0.24	TCMSP
(2R)-5,7-dihydroxy-2-(4-hydroxyphenyl)chroman-4-one	42.36	0.21	TCMSP
11-Hydroxynumantenine	50.79	0.71	TCMSP
Harmonyl	43.8	0.46	TCMSP
ZINC00035529	58.39	0.22	TCMSP

Flavidin	30.1	0.26	TCMSP
Albaspidin AA	31.16	0.36	TCMSP
Isoramanone	39.97	0.51	TCMSP
1-methyl-2-nonacosyl-4-quinolone	31.54	0.5	TCMSP
Ruvoside_qt	36.12	0.76	TCMSP
C09747	37.28	0.25	TCMSP
Diop	43.59	0.39	TCMSP
patchoulan 1,12-diol	38.17	0.25	TCMSP
pachypodol	75.06	0.4	TCMSP
5-Hydroxy-7,4'-dimethoxyflavanon	51.54	0.27	TCMSP
irisolidone	37.78	0.3	TCMSP
phenanthrone	38.7	0.33	TCMSP
quercetin 7-O-β-D-glucoside	49.57	0.27	TCMSP
Acanthoside B	43.35	0.77	TCMSP
3,23-dihydroxy-12-oleanen-28-oic acid	30.86	0.86	TCMSP
EUPATIN	50.8	0.41	TCMSP
Mutatochrome	48.64	0.61	TCMSP
Physciondiglucoside	41.65	0.63	TCMSP
Procyanidin B-5,3'-O-gallate	31.99	0.32	TCMSP
rhein	47.07	0.28	TCMSP
Sennoside E_qt	50.69	0.61	TCMSP
Torachrysone-8-O-beta-D-(6'-oxayl)-glucoside	43.02	0.74	TCMSP
Toralactone	46.46	0.24	TCMSP
Emodin-1-O-beta-D-glucopyranoside	44.81	0.8	TCMSP
Sennoside D_qt	61.06	0.61	TCMSP
Daucosterol_qt	35.89	0.7	TCMSP
palmidin A	32.45	0.65	TCMSP
aloe-emodin	83.38	0.24	TCMSP
gallic acid-3-O-(6'-O-galloyl)-glucoside	30.25	0.67	TCMSP
(-)-catechin	49.68	0.24	TCMSP
Inermine	75.18	0.54	TCMSP
Jaranol	50.83	0.29	TCMSP
Medicarpin	49.22	0.34	TCMSP
isorhamnetin	49.6	0.31	TCMSP
Lupiwighteone	51.64	0.37	TCMSP
7-Methoxy-2-methyl isoflavone	42.56	0.2	TCMSP
formononetin	69.67	0.21	TCMSP
Calycosin	47.75	0.24	TCMSP
(2S)-2-[4-hydroxy-3-(3-methylbut-2-enyl)phenyl]-8,8-dimethyl-2,3-dihydropyrano[2,3-f]chromen-4-one	31.79	0.72	TCMSP
euchrenone	30.29	0.57	TCMSP

glyasperin B	65.22	0.44	TCMSP
glyasperin F	75.84	0.54	TCMSP
Glyasperin C	45.56	0.4	TCMSP
Isotrifoliol	31.94	0.42	TCMSP
(E)-1-(2,4-dihydroxyphenyl)-3-(2,2-dimethylchromen-6-yl)prop-2-en-1-one	39.62	0.35	TCMSP
kanzonols W	50.48	0.52	TCMSP
(2S)-6-(2,4-dihydroxyphenyl)-2-(2-hydroxypropan-2-yl)-4-methoxy-2,3-dihydrofuro[3,2-g]chromen-7-one	60.25	0.63	TCMSP
Semilicoisoflavone B	48.78	0.55	TCMSP
Glepidotin A	44.72	0.35	TCMSP
Glepidotin B	64.46	0.34	TCMSP
Phaseolinisoflavan	32.01	0.45	TCMSP
Glypallichalcone	61.6	0.19	TCMSP
8-(6-hydroxy-2-benzofuranyl)-2,2-dimethyl-5-chromenol	58.44	0.38	TCMSP
licochalcone G	49.25	0.32	TCMSP
3-(2,4-dihydroxyphenyl)-8-(1,1-dimethylprop-2-enyl)-7-hydroxy-5-methoxy-coumarin	59.62	0.43	TCMSP
Licoricone	63.58	0.47	TCMSP
Gancaonin A	51.08	0.4	TCMSP
Gancaonin B	48.79	0.45	TCMSP
licorice glycoside E	32.89	0.27	TCMSP
3-(3,4-dihydroxyphenyl)-5,7-dihydroxy-8-(3-methylbut-2-enyl)chromone	66.37	0.41	TCMSP
5,7-dihydroxy-3-(4-methoxyphenyl)-8-(3-methylbut-2-enyl)chromone	30.49	0.41	TCMSP
2-(3,4-dihydroxyphenyl)-5,7-dihydroxy-6-(3-methylbut-2-enyl)chromone	44.15	0.41	TCMSP
Glycyrin	52.61	0.47	TCMSP
Licocoumarone	33.21	0.36	TCMSP
Licoisoflavone	41.61	0.42	TCMSP
Licoisoflavone B	38.93	0.55	TCMSP
licoisoflavanone	52.47	0.54	TCMSP
shinpterocarpin	80.3	0.73	TCMSP
(E)-3-[3,4-dihydroxy-5-(3-methylbut-2-enyl)phenyl]-1-(2,4-dihydroxyphenyl)prop-2-en-1-one	46.27	0.31	TCMSP
licopyranocoumarin	80.36	0.65	TCMSP
3,22-Dihydroxy-11-oxo-delta(12)-oleanene-27-alpha-methoxycarbonyl-29-oic acid	34.32	0.55	TCMSP
Glyzaglabrin	61.07	0.35	TCMSP
Glabranin	52.9	0.31	TCMSP

Glabrene	46.27	0.44	TCMSP
Glabrone	52.51	0.5	TCMSP
1,3-dihydroxy-9-methoxy-6-benzofurano[3,2-c]chromenone	48.14	0.43	TCMSP
1,3-dihydroxy-8,9-dimethoxy-6-benzofurano[3,2-c]chromenone	62.9	0.53	TCMSP
Eurycarpin A	43.28	0.37	TCMSP
glycyroside	37.25	0.79	TCMSP
(-)-Medicocarpin	40.99	0.95	TCMSP
Sigmoidin-B	34.88	0.41	TCMSP
(2R)-7-hydroxy-2-(4-hydroxyphenyl)chroman-4-one	71.12	0.18	TCMSP
(2S)-7-hydroxy-2-(4-hydroxyphenyl)-8-(3-methylbut-2-enyl)chroman-4-one	36.57	0.32	TCMSP
Isoglycyrol	44.7	0.84	TCMSP
Isolicoflavonol	45.17	0.42	TCMSP
HMO	38.37	0.21	TCMSP
1-Methoxyphaseollidin	69.98	0.64	TCMSP
Quercetin der.	46.45	0.33	TCMSP
3'-Hydroxy-4'-O-Methylglabridin	43.71	0.57	TCMSP
licochalcone a	40.79	0.29	TCMSP
3'-Methoxyglabridin	46.16	0.57	TCMSP
2-[(3R)-8,8-dimethyl-3,4-dihydro-2H-pyrano[6,5-f]chromen-3-yl]-5-methoxyphenol	36.21	0.52	TCMSP
Inflacoumarin A	39.71	0.33	TCMSP
icos-5-enoic acid	30.7	0.2	TCMSP
Kanzonol F	32.47	0.89	TCMSP
6-prenylated eriodictyol	39.22	0.41	TCMSP
7,2',4'-trihydroxy－5-methoxy-3－arylcoumarin	83.71	0.27	TCMSP
7-Acetoxy-2-methylisoflavone	38.92	0.26	TCMSP
8-prenylated eriodictyol	53.79	0.4	TCMSP
gadelaidic acid	30.7	0.2	TCMSP
Vestitol	74.66	0.21	TCMSP
Gancaonin G	60.44	0.39	TCMSP
Gancaonin H	50.1	0.78	TCMSP
Licoagrocarpin	58.81	0.58	TCMSP
Glyasperins M	72.67	0.59	TCMSP
Glycyrrhiza flavonol A	41.28	0.6	TCMSP
Licoagroisoflavone	57.28	0.49	TCMSP
18α-hydroxyglycyrrhetic acid	41.16	0.71	TCMSP
Odoratin	49.95	0.3	TCMSP
Xambioona	54.85	0.87	TCMSP
dehydroglyasperins C	53.82	0.37	TCMSP
caso4·2h2o	-	-	TCMID

Menthol	-	-	Batman
salidroside	-	-	Batman

Supplementary Table 3: The related targets and gene symbols of the ingredients in LHQW-C.

Ingredient	Target	Symbol
wogonin	Nitric oxide synthase, inducible	NOS2
wogonin	Prostaglandin G/H synthase 1	PTGS1
wogonin	Estrogen receptor	ESR1
wogonin	Androgen receptor	AR
wogonin	Sodium channel protein type 5 subunit alpha	SCN5A
wogonin	Peroxisome proliferator activated receptor gamma	PPARG
wogonin	Prostaglandin G/H synthase 2	PTGS2
wogonin	Retinoic acid receptor RXR-alpha	RXRA
wogonin	CGMP-inhibited 3',5'-cyclic phosphodiesterase A	PDE3A
wogonin	Dipeptidyl peptidase IV	DPP4
wogonin	Mitogen-activated protein kinase 14	MAPK14
wogonin	Glycogen synthase kinase-3 beta	GSK3B
wogonin	Phosphatidylinositol-4,5-bisphosphate 3-kinase catalytic subunit, gamma isoform	PIK3CG
wogonin	Serine/threonine-protein kinase Chk1	CHEK1
wogonin	Trypsin-1	PRSS1
wogonin	Calmodulin	CAM
wogonin	Beta-2 adrenergic receptor	ADRB2
wogonin	Gamma-aminobutyric acid receptor subunit alpha-1	GABRA1
wogonin	Transcription factor p65	RELA
wogonin	RAC-alpha serine/threonine-protein kinase	AKT1
wogonin	G1/S-specific cyclin-D1	CCND1
wogonin	Apoptosis regulator Bcl-2	BCL2
wogonin	Cyclin-dependent kinase inhibitor 1	CDKN1A
wogonin	Eukaryotic translation initiation factor 6	EIF6
wogonin	Apoptosis regulator BAX	BAX
wogonin	Caspase-9	CASP9
wogonin	Vascular endothelial growth factor receptor 2	KDR
wogonin	Tumor necrosis factor	TNF
wogonin	Transcription factor AP-1	JUN
wogonin	Interleukin-6	IL6

wogonin	Activator of 90 kDa heat shock protein ATPase homolog 1	AHSA1
wogonin	Caspase-3	CASP3
wogonin	Cellular tumor antigen p53	TP63
wogonin	Bcl-2-binding component 3	BBC3
wogonin	Telomerase protein component 1	TEP1
wogonin	Interstitial collagenase	MMP1
wogonin	C-C motif chemokine 2	CCL2
wogonin	Protein kinase C delta type	PRKCD
wogonin	Prostaglandin E2 receptor EP3 subtype	PTGER3
wogonin	Fibronectin	FN1
wogonin	Interleukin-8	CXCL8
wogonin	Induced myeloid leukemia cell differentiation protein Mcl-1	MCL1
(2R,3R,4S)-4-(4-hydroxy-3-methoxy-phenyl)-7-methoxy-2,3-dimethylol-tetralin-6-ol	Nitric-oxide synthase, endothelial	NOS3
(2R,3R,4S)-4-(4-hydroxy-3-methoxy-phenyl)-7-methoxy-2,3-dimethylol-tetralin-6-ol	Carbonic anhydrase II	CA2
(2R,3R,4S)-4-(4-hydroxy-3-methoxy-phenyl)-7-methoxy-2,3-dimethylol-tetralin-6-ol	Coagulation factor VII	F7
(2R,3R,4S)-4-(4-hydroxy-3-methoxy-phenyl)-7-methoxy-2,3-dimethylol-tetralin-6-ol	DNA topoisomerase II	TOP2B
(2R,3R,4S)-4-(4-hydroxy-3-methoxy-phenyl)-7-methoxy-2,3-dimethylol-tetralin-6-ol	Estrogen receptor beta	ESR2
(2R,3R,4S)-4-(4-hydroxy-3-methoxy-phenyl)-7-methoxy-2,3-dimethylol-tetralin-6-ol	Proto-oncogene serine/threonine-protein kinase Pim-1	PIM1
(2R,3R,4S)-4-(4-hydroxy-3-methoxy-phenyl)-7-methoxy-2,3-dimethylol-tetralin-6-ol	Nuclear receptor coactivator 2	NCOA2
(2R,3R,4S)-4-(4-hydroxy-3-methoxy-phenyl)-7-methoxy-2,3-dimethylol-tetralin-6-ol	Cyclin-A2	CCNA2
(2R,3R,4S)-4-(4-hydroxy-3-methoxy-phenyl)-7-methoxy-2,3-dimethylol-tetralin-6-ol	Ig gamma-1 chain C region	IGHG1

(3R,4R)-3,4-bis[(3,4-dimethoxyphenyl)methyl]oxolan-2-one	Muscarinic acetylcholine receptor M3	CHRM3
(3R,4R)-3,4-bis[(3,4-dimethoxyphenyl)methyl]oxolan-2-one	Potassium voltage-gated channel subfamily H member 2	KCNH2
(3R,4R)-3,4-bis[(3,4-dimethoxyphenyl)methyl]oxolan-2-one	Alpha-1B adrenergic receptor	ADRA1B
(3R,4R)-3,4-bis[(3,4-dimethoxyphenyl)methyl]oxolan-2-one	mRNA of Protein-tyrosine phosphatase, non-receptor type 1	PTPN1
(3R,4R)-3,4-bis[(3,4-dimethoxyphenyl)methyl]oxolan-2-one	Sodium-dependent dopamine transporter	SLC6A3
(3R,4R)-3,4-bis[(3,4-dimethoxyphenyl)methyl]oxolan-2-one	Alpha-1D adrenergic receptor	ADRA1D
(+)-pinoresinol monomethyl ether	Retinoic acid receptor RXR-beta	RXRB
(+)-pinoresinol monomethyl ether	Nuclear receptor coactivator 1	NCOA1
3beta-Acetyl-20,25-epoxydammarane-24alpha-ol	Glucocorticoid receptor	NR3C1
Mairin	Progesterone receptor	PGR
(-)-Phillygenin	Muscarinic acetylcholine receptor M1	CHRM1
(-)-Phillygenin	Muscarinic acetylcholine receptor M5	CHRM5
hyperforin	Cytochrome P450 3A4	CYP3A4
hyperforin	Intercellular adhesion molecule 1	ICAM1
hyperforin	Nuclear receptor subfamily 1 group I member 2	NR1I2
Onjixanthone I	Calcium-activated potassium channel subunit alpha 1	KCNMA1
beta-sitosterol	Gamma-aminobutyric-acid receptor alpha-2 subunit	GABRA2
beta-sitosterol	Muscarinic acetylcholine receptor M4	CHRM4
beta-sitosterol	5-hydroxytryptamine 2A receptor	HTR2A
beta-sitosterol	Gamma-aminobutyric-acid receptor alpha-5 subunit	GABRA5
beta-sitosterol	Alpha-1A adrenergic receptor	ADRA1A

beta-sitosterol	Gamma-aminobutyric-acid receptor alpha-3 subunit	GABRA3
beta-sitosterol	Muscarinic acetylcholine receptor M2	CHRM2
beta-sitosterol	Neuronal acetylcholine receptor subunit alpha-2	CHRNA2
beta-sitosterol	Sodium-dependent serotonin transporter	SLC6A4
beta-sitosterol	Mu-type opioid receptor	OPRM1
beta-sitosterol	Neuronal acetylcholine receptor protein, alpha-7 chain	CHRNA7
beta-sitosterol	Caspase-8	CASP8
beta-sitosterol	Protein kinase C alpha type	PRKCA
beta-sitosterol	Transforming growth factor beta-1	TGFB1
beta-sitosterol	Serum paraoxonase/arylesterase 1	PON1
beta-sitosterol	Microtubule-associated protein 2	MAP2
kaempferol	Acetylcholinesterase	ACHE
kaempferol	Sodium-dependent noradrenaline transporter	SLC6A2
kaempferol	Inhibitor of nuclear factor kappa-B kinase subunit beta	IKBKB
kaempferol	Mitogen-activated protein kinase 8	MAPK8
kaempferol	Xanthine dehydrogenase/oxidase	XDH
kaempferol	Signal transducer and activator of transcription 1-alpha/beta	STAT1
kaempferol	Heme oxygenase 1	HMOX1
kaempferol	Cytochrome P450 1A2	CYP1A2
kaempferol	Cytochrome P450 1A1	CYP1A1
kaempferol	E-selectin	SELE
kaempferol	Vascular cell adhesion protein 1	VCAM1
kaempferol	Cytochrome P450 1B1	CYP1B1
kaempferol	Arachidonate 5-lipoxygenase	ALOX5
kaempferol	Hyaluronan synthase 2	HAS2
kaempferol	Glutathione S-transferase P	GSTP1
kaempferol	Aryl hydrocarbon receptor	AHR
kaempferol	26S proteasome non-ATPase regulatory subunit 3	PSMD3
kaempferol	Solute carrier family 2, facilitated glucose transporter member 4	SLC2A4
kaempferol	Nuclear receptor subfamily 1 group I member 3	NR1I3
kaempferol	Insulin receptor	INSR
kaempferol	Type I iodothyronine deiodinase	DIO1

kaempferol	Serine/threonine-protein phosphatase 2B catalytic subunit alpha isoform	PPP3CA
kaempferol	Glutathione S-transferase Mu 1	GSTM1
kaempferol	Glutathione S-transferase Mu 2	GSTM2
kaempferol	Aldo-keto reductase family 1 member C3	AKR1C3
kaempferol	Antileukoproteinase	SLPI
arctiin	Mucin-1	MUC1
luteolin	Epidermal growth factor receptor	EGFR
luteolin	Vascular endothelial growth factor A	VEGFA
luteolin	Bcl-2-like protein 1	BCL2L1
luteolin	72 kDa type IV collagenase	MMP2
luteolin	Matrix metalloproteinase-9	MMP9
luteolin	Mitogen-activated protein kinase 1	MAPK1
luteolin	Interleukin-10	IL10
luteolin	Retinoblastoma-associated protein	RB1
luteolin	NF-kappa-B inhibitor alpha	NFKBIA
luteolin	DNA topoisomerase 1	TOP1
luteolin	E3 ubiquitin-protein ligase Mdm2	MDM2
luteolin	Amyloid beta A4 protein	APP
luteolin	Proliferating cell nuclear antigen	PCNA
luteolin	Receptor tyrosine-protein kinase erbB-2	ERBB2
luteolin	Caspase-7	CASP7
luteolin	Baculoviral IAP repeat-containing protein 5	BIRC5
luteolin	Interleukin-2	IL2
luteolin	G2/mitotic-specific cyclin-B1	CCNB1
luteolin	Tyrosinase	TYR
luteolin	Interferon gamma	IFNG
luteolin	Interleukin-4	IL4
luteolin	DNA topoisomerase 2-alpha	TOP2A
luteolin	CD40 ligand	CD40LG
luteolin	Prostaglandin E synthase	PTGES
luteolin	Kinetochore protein Nuf2	NUF2
luteolin	Adenylate cyclase type 2	ADCY2
luteolin	Hepatocyte growth factor receptor	MET
bicuculline	Proto-oncogene c-Fos	FOS
bicuculline	Gap junction alpha-1 protein	GJA1
bicuculline	Gamma-aminobutyric acid type B receptor subunit 1	GABBR1

bicuculline	Bone morphogenetic protein receptor type-2	BMPR2
bicuculline	Metabotropic glutamate receptor 5	GRM5
bicuculline	Progonadoliberin-1	GNRH1
bicuculline	Aldehyde dehydrogenase, dimeric NADP-preferring	ALDH3A1
bicuculline	Gonadotropin-releasing hormone receptor	GNRHR
bicuculline	Corticoliberin	CRH
bicuculline	Gap junction beta-1 protein	GJB1
bicuculline	Metabotropic glutamate receptor 1	GRM1
bicuculline	Transitional endoplasmic reticulum ATPase	VCP
quercetin	Aldose reductase	AKR1B1
quercetin	Stromelysin-1	MMP3
quercetin	Amine oxidase [flavin-containing] B	MAOB
quercetin	Urokinase-type plasminogen activator	PLAU
quercetin	Pro-epidermal growth factor	EGF
quercetin	ETS domain-containing protein Elk-1	ELK1
quercetin	NADPH--cytochrome P450 reductase	POR
quercetin	Ornithine decarboxylase	ODC1
quercetin	RAF proto-oncogene serine/threonine-protein kinase	RAF1
quercetin	Superoxide dismutase [Cu-Zn]	SOD1
quercetin	Hypoxia-inducible factor 1-alpha	HIF1A
quercetin	Protein CBFA2T1	RUNX1T1
quercetin	Acetyl-CoA carboxylase 1	ACACA
quercetin	Caveolin-1	CAV1
quercetin	Myc proto-oncogene protein	MYC
quercetin	Tissue factor	F3
quercetin	Interleukin-1 beta	IL1B
quercetin	Protein kinase C beta type	PRKCB
quercetin	Dual oxidase 2	DUOX2
quercetin	Heat shock protein beta-1	HSPB1
quercetin	Estrogen sulfotransferase	SULT1E1
quercetin	Maltase-glucoamylase, intestinal	MGAM
quercetin	Tissue-type plasminogen activator	PLAT
quercetin	Thrombomodulin	THBD
quercetin	Plasminogen activator inhibitor 1	SERPINE1
quercetin	Collagen alpha-1(I) chain	COL1A1

quercetin	Phosphatidylinositol-3,4,5-trisphosphate 3-phosphatase and dual-specificity protein phosphatase PTEN	PTENPTEN
quercetin	Interleukin-1 alpha	IL1A
quercetin	Myeloperoxidase	MPO
quercetin	Neutrophil cytosol factor 1	NCF1
quercetin	ATP-binding cassette sub-family G member 2	ABCG2
quercetin	Nuclear factor erythroid 2-related factor 2	NFE2L2
quercetin	NAD(P)H dehydrogenase [quinone] 1	NQO1
quercetin	Poly [ADP-ribose] polymerase 1	PARP1
quercetin	Collagen alpha-1(III) chain	COL3A1
quercetin	C-X-C motif chemokine 11	CXCL11
quercetin	C-X-C motif chemokine 2	CXCL2
quercetin	DDB1- and CUL4-associated factor 5	DCAF5
quercetin	Serine/threonine-protein kinase Chk2	CHEK2
quercetin	Claudin-4	CLDN4
quercetin	Peroxisome proliferator-activated receptor alpha	PPARA
quercetin	Peroxisome proliferator-activated receptor delta	PPARD
quercetin	Heat shock factor protein 1	HSF1
quercetin	C-reactive protein	CRP
quercetin	C-X-C motif chemokine 10	CXCL10
quercetin	Inhibitor of nuclear factor kappa-B kinase subunit alpha	CHUK
quercetin	Osteopontin	SPP1
quercetin	Runt-related transcription factor 2	RUNX2
quercetin	Ras association domain-containing protein 1	RASSF1
quercetin	Transcription factor E2F1	E2F1
quercetin	Transcription factor E2F2	E2F2
quercetin	Prostatic acid phosphatase	ACPP
quercetin	Cathepsin D	CTSD
quercetin	Insulin-like growth factor-binding protein 3	IGFBP3
quercetin	Insulin-like growth factor II	IGF2
quercetin	Interferon regulatory factor 1	IRF1
quercetin	Receptor tyrosine-protein kinase erbB-3	ERBB3
quercetin	Procollagen C-endopeptidase enhancer 1	PCOLCE

quercetin	Puromycin-sensitive aminopeptidase	NPEPPS
quercetin	Hexokinase-2	HK2
quercetin	Homeobox protein Nkx-3.1	NKX3-1
Eriodyctiol (flavanone)	Glycogen phosphorylase, muscle form	PYGM
beta-carotene	Serum albumin	ALB
beta-carotene	Catenin beta-1	CTNNB1
beta-carotene	Stromelysin-2	MMP10
ZINC03978781	Mineralocorticoid receptor	NR3C2
5-hydroxy-7-methoxy-2-(3,4,5-trimethoxyphenyl)chromone	Voltage-dependent calcium channel subunit alpha-2/delta-1	CACNA2D1
Stigmasterol	Alcohol dehydrogenase 1C	ADH1C
Stigmasterol	Alpha-2A adrenergic receptor	ADRA2A
Stigmasterol	Leukotriene A-4 hydrolase	LTA4H
Stigmasterol	Amine oxidase [flavin-containing] A	MAOA
Stigmasterol	Chymotrypsinogen B	CTRB1
Stigmasterol	Beta-1 adrenergic receptor	ADRB1
quercetin	Ras GTPase-activating protein 1	RASA1
(+)-catechin	Catalase	CAT
naringenin	Mitogen-activated protein kinase 3	MAPK3
naringenin	Fatty acid synthase	FASN
naringenin	Low-density lipoprotein receptor	LDLR
naringenin	Bcl2 antagonist of cell death	BAD
naringenin	Microsomal triglyceride transfer protein large subunit	MTTP
naringenin	Apolipoprotein B-100	APOB
naringenin	Phospholipase B1, membrane-associated	PLB1
naringenin	3-hydroxy-3-methylglutaryl-coenzyme A reductase	HMGCR
naringenin	UDP-glucuronosyltransferase 1-1	UGT1A1
naringenin	Sterol regulatory element-binding protein 1	SREBF1
naringenin	Glutathione reductase, mitochondrial	GSR
naringenin	Multidrug resistance-associated protein 1	ABCC1
naringenin	Adiponectin	ADIPOQ
naringenin	Sterol O-acyltransferase 2	SOAT2
naringenin	Aldo-keto reductase family 1 member C1	AKR1C1
naringenin	Aspartate aminotransferase, cytoplasmic	GOT1
naringenin	4-aminobutyrate aminotransferase, mitochondrial	ABAT
naringenin	Liver carboxylesterase 1	CES1

naringenin	Sterol O-acyltransferase 1	SOAT1
taxifolin	Diacylglycerol O-acyltransferase 2	DGAT2
estrone	Delta-type opioid receptor	OPRD1
estrone	5-hydroxytryptamine 2C receptor	HTR2C
estrone	Kappa-type opioid receptor	OPRK1
estrone	D(2) dopamine receptor	DRD2
Machiline	Alpha-2C adrenergic receptor	ADRA2C
Machiline	Alpha-2B adrenergic receptor	ADRA2B
l-SPD	D(1B) dopamine receptor	DRD5
l-SPD	D(3) dopamine receptor	DRD3
acacetin	Tumor necrosis factor ligand superfamily member 6	FASLG
Dinatin	Rhodopsin	RHO
quindoline	cAMP-dependent protein kinase inhibitor alpha	PKIA
aloe-emodin	Protein kinase C epsilon type	PRKCE
(-)-catechin	Krueppel-like factor 7	KLF7
Inermine	5-hydroxytryptamine receptor 3A	HTR3A
Medicarpin	Mitogen-activated protein kinase 10	MAPK10
isorhamnetin	Glutamate receptor 2	GRIA2
isorhamnetin	Oxidized low-density lipoprotein receptor 1	OLR1
formononetin	NAD-dependent deacetylase sirtuin-1	SIRT1
formononetin	ATP synthase subunit beta, mitochondrial	ATP5F1B
formononetin	NADH-ubiquinone oxidoreductase chain 6	ND6
formononetin	3 beta-hydroxysteroid dehydrogenase/Delta 5-->4-isomerase type 2	HSD3B2
formononetin	3 beta-hydroxysteroid dehydrogenase/Delta 5-->4-isomerase type 1	HSD3B1
licochalcone a	Signal transducer and activator of transcription 3	STAT3
licochalcone a	Fos-related antigen 2	FOSL2
Menthol	Cholinesterase	BCHE

Supplementary Table 4: 643 COVID-19-related genes collected from GeneCards.

ACE2	AASS	POLA2	ARL6IP6	IFITM1	JAK1	PRKRA
DPP4	BRD2	SRP72	AAR2	IFITM2	EIF2AK3	FGF7
IL2RA	BRD4	UPF1	CEP350	TP53	CXCL2	SLPI
LOC117134608	MFGE8	RAB14	DNAJC11	CCND1	IL18	TERF1
LOC117135106	LOX	RAB18	EDEM3	VIM	BAG3	MPP5
CTSL	CYB5R3	STC2	GRIPAP1	ADAM17	IL13	CPNE3
TMPRSS2	NEU1	RAB8A	DCAF7	RB1	HSPB1	DCTN2
LOC117134604	IL17RA	RAE1	EMC1	CDK4	EEF1A1	BTF3
LOC117134605	RTN4	TMPRSS4	RRP9	BCL2	CCNA2	PPIG
LOC117134606	NGLY1	ATE1	NSD2	SGTA	MKRN3	FBL
LOC117134607	POFUT1	EXOSC5	TRIM59	CCR5	SMAD4	MYOM2
LOC117134611	TOR1A	ALG5	UBAP2L	NFKB1	APOBEC3G	SLC1A5
LOC117135104	PVR	BZW2	TBKBP1	CXCR3	CCR3	PSMA2
LOC117135105	TLE1	HOOK1	TIMM10B	MAPK14	ANXA2	SPEN
IL6	PRKAR2B	GIGYF2	USP54	MAPK8	CANX	BAG6
CD8A	USP13	ANO6	ZC3H7A	CASP3	PML	SPIDR
GPT	AKAP9	GRPEL1	ZNF503	CDK6	KPNB1	HGS
AGTR2	AP2M1	DDX10	ZNF318	IL5	NOS3	NPIPB3
FURIN	AGPS	RNF41	YIF1A	MAPK1	SOCS3	MARK2
ACE	COL6A1	CRTC3	CCDC86	CLEC12A	SERPINE1	RPS20
CRP	NUP214	SDF2	FBXL12	SMAD3	KPNA2	PFKP
INS	SEPSECS	RPL36	FKBP15	AHSG	PSMC6	EIF4B
LOC117134593	SRP54	NUP210	HSBP1	FGL2	IKBKE	PPIB
ENPEP	PTGES2	PITRM1	TYSND1	PRKCA	NLRP3	MNAT1
LOC117152610	RAB2A	SNIP1	NAT14	C17orf49	KRT8	KMT2B
LOC117152611	TARS2	SLC30A7	ZYG11B	IL1A	TUBA1B	TPSAB1

LOC117204000	RAB10	NUP88	FAM8A1	PKLR	TTR	NPEPPS
LOC117204001	TLE3	PSMD8	MEPCE	DDIT3	ISG15	PLA2G2D
LOC117600004	NARS2	TOR1AIP1	FOXRED2	IKBKB	EIF4E	DDAH2
LOC117693187	HYOU1	REEP5	CLIP4	MASP2	MAVS	BCL2A1
KNG1	PMPCA	REEP6	ELOC	UBE2I	TMEM233	UBB
IL10	EXOSC3	STOM	PRRC2B	PARP1	RELB	MCRS1
HLA-A	CDK5RAP2	MYCBP2	NGDN	TGFB1	SFTPD	MARK3
CTSB	CENPF	RBM28	LARP4B	CCND3	MCL1	RPS17
VEGFA	GGH	VPS39	RBM41	IFTAP	STING1	RFC2
BSG	NUP98	HECTD1	TM2D3	IRF1	PHB	SNRNP48
EGFR	RBX1	CEP135	NOL10	XBP1	THPO	DIDO1
IL6R	CUL2	MRPS2	MAP7D1	DDX5	EIF2AK4	PFDN5
HLA-DRB1	PMPCB	DDX21	ERO1B	ITGAL	KRT19	ATP6V1G1
PLAUR	GDF15	LARP1	FASTKD5	IL1B	IFNL1	CHMP2B
CD209	EXOSC2	LMAN2	COQ8B	MAPK3	CCR1	FRY
IL17A	ATP6AP1	MDN1	ELOB	JPH4	VDAC1	CRB3
CD14	FBN2	GCC2	SAAL1	BAX	POLR2A	RYBP
EZH2	ERC1	GOLGB1	UBAP2	FCER2	DNAJB1	PRSS2
HMOX1	ERP44	AP2A2	PUSL1	CASP8	F3	NOMO1
COMT	CYB5B	ATP13A3	ZC3H18	ATF6	TRIM25	CAPRIN1
HDAC2	GTF2F2	CHPF	FAM98A	EIF2AK2	HELLS	CCHCR1
PRKACA	DNAJC19	MRPS25	HEATR3	CXCL16	STAT2	RCAN3
DNMT1	EIF4H	MTCH1	CEP112	BCL2L1	FKBP1A	PLEKHO1
ITGB1	NPC2	MPHOSPH10	SPART	CDK2	GZMA	CAMLG
RIPK1	PABPC1	PDZD11	SELENOS	CREB1	STAT6	NPHP3
TBK1	PABPC4	NPTX1	ATP5MG	EZR	USP7	TPSB2
PLAT	PCNT	SLU7	CISD3	SFTPC	SERPING1	ARL4D
GLA	NIN	SRP19	TLE5	CSF2	TUBB3	BCL2L2
GPX1	PLD3	INHBE	WASHC4	HLA-B	PSMC1	SNAP47

IDE	SLC30A9	TUBGCP2	C1orf50	CREBBP	CCNE1	FAHD1
RDX	SLC25A21	TUBGCP3	CEP43	SDCBP	YWHAE	MACROH2A2
RAB7A	SIL1	UGGT2	NUP58	GAPDH	ENO1	BRF1
POR	LARP7	JAKMIP1	MTARC1	SUMO1	PYCARD	ATF5
ATP1B1	RAP1GDS1	SUN2	POGLUT3	IFI30	RCHY1	RPP38
ADAM9	RAB1A	PPIL3	POGLUT2	LOC117307477	UBD	PPIH
ACADM	RAB5C	THTPA	TIMM29	HNRNPA1	ADAM10	SARS1
CSNK2A2	PRIM1	TIMM8B	SLC6A19	FOS	FCGR2A	NMB
CSNK2B	PRIM2	TIMM9	FUT3	PPIA	KPNA1	NAE1
IMPDH2	TRMT1	ZDHHC5	ANPEP	BAD	PI4KB	TBCB
TCF12	MOGS	AKAP8L	CLEC4G	RUNX1	IGKC	MIF4GD
GGCX	VPS11	GCC1	TNF	HSPA5	TIAM1	N4BP2L2
RHOA	AKAP8	ERLEC1	IFNA1	CASP6	PHB2	ZCRB1
PRKAR2A	MAT2B	FKBP7	IFNG	NPM1	PCSK7	MT-ND4L
POLA1	GOLGA2	G3BP2	CCL2	PKD1	PCM1	C20orf27
SIGMAR1	GNG5	CEP68	CXCL8	BAK1	VKORC1	MKRN2
MIB1	GORASP1	GOLGA7	IFNB1	MAPKAPK2	XPA	HOXC6
GNB1	EXOSC8	CLCC1	STAT1	VHL	CAMK2D	ISLR
AP3B1	ALG11	COLGALT1	IL2	TRAF6	LDHA	TAP1
FBN1	ERGIC1	CHPF2	IFIH1	RPS27A	TLR10	ISG20
BCKDK	FKBP10	MRPS27	CXCL10	TRAF2	UBQLN1	RNF128
GFER	AATF	MRPS5	CLEC4M	ALB	LAS1L	DEDD2
ETFA	ACSL3	DCAKD	CCL5	HLA-C	STOML2	NOMO3
CIT	FYCO1	SCCPDH	SH2D3A	HSP90B1	MT-CO2	SLC46A3
NEK9	G3BP1	SBNO1	CCL3	IFN1@	H1-1	NCOA5
SCARB1	CWC27	NINL	CXCL9	BAP1	CKAP5	NDUFA10
PKP2	HS2ST1	PLEKHA5	IRF3	EP300	TMPRSS11A	NUDT2
PLOD2	HS6ST2	PLEKHF2	DDX1	BMP6	RNF123	OAS1
SIRT5	CEP250	NUP54	MBL2	ACTB	IL16	ABHD17A
SLC27A2	MOV10	NUTF2	MX1	CHEK2	NMI	OCIAD2
NUP62	NDUFAF2	INTS4	CD40LG	ATF2	HPN	C1orf162
RALA	EIF4E2	QSOX2	TRAF3	UBC	POLR2B	IFNAR1
PPT1	PCSK6	CNTRL	DDX58	CDKN1B	BID	ZNF410

ARF6	PDE4DIP	TIMM10	TMPRSS11D	EIF2S1	EIF3M	OR8U9
ATP6V1A	SLC44A2	MIPOL1	ICAM3	EGR1	TMEM43	C8G
FBLN5	SMOC1	GOLGA3	CTRL	IL4	SNAPIN	RSRP1
ADAMTS1	OS9	FAM162A	IFITM3	CD79A	LCP1	

Supplementary Table 5: The 49 overlapping gene symbols between COVID-19 and LHQW-C.

DPP4	CASP3	PRKCA	EGFR	IFNG	HSPB1	CRP
MAPK14	CCL2	TGFB1	VEGFA	IL4	PLAT	CXCL10
CCND1	CXCL8	IKBKB	BCL2L1	CD40LG	SERPINE1	IRF1
BCL2	MCL1	MAPK8	MAPK1	FOS	IL1A	NPEPPS
BAX	NOS3	STAT1	IL10	POR	PARP1	ALB
TNF	CCNA2	HMOX1	RB1	F3	CXCL2	MAPK3
IL6	CASP8	SLPI	IL2	IL1B	CHEK2	BAD

Supplementary Table 6: The top 20 biological process (BP), molecular function (MF), and cellular component (CC) terms.

ONTOLOGY	ID	Description	pvalue	p.adjust	geneID	Count
BP	GO:0032496	response to lipopolysaccharide	2.38977E-29	7.66637E-26	NOS2/PTGS2/FOS/CASP9/MAPK1/IL10/JUN/IL6/CASP3/NFKBIA/CASP8/CYP1A2/GJA1/CYP1A1/ICAM1/IL1B/CCL2/SELE/VCAM1/CXCL8/NOS3/THBD/SERPINE1/MPO/GSTP1/CXCL2/CHUK	27
BP	GO:0002237	response to molecule of bacterial origin	6.86823E-29	1.10166E-25	NOS2/PTGS2/FOS/CASP9/MAPK1/IL10/JUN/IL6/CASP3/NFKBIA/CASP8/CYP1A2/GJA1/CYP1A1/ICAM1/IL1B/CCL2/SELE/VCAM1/CXCL8/NOS3/THBD/SERPINE1/MPO/GSTP1/CXCL2/CHUK	27
BP	GO:0010038	response to metal ion	1.55629E-19	1.66419E-16	PTGS2/FOS/CASP9/MMP9/MAPK1/JUN/CASP3/CASP8/SOD1/HIF1A/CYP1A2/CAV1/CYP1A1/ICAM1/VCAM1/CCNB1/IL1A/NFE2L2/NQO1/PARP1/CHUK	21
BP	GO:0072593	reactive oxygen species metabolic process	6.74235E-19	5.40737E-16	NOS2/PTGS2/CDKN1A/IL10/SOD1/HIF1A/CYP1A2/CAV1/CYP1A1/ICAM1/IL1B/DUOX2/NOS3/IFNG/	19

					MPO/GSTP1/NFE2L2/NQO1/CRP	
BP	GO:0006979	response to oxidative stress	1.25356E-17	8.04286E-15	PTGS1/PTGS2/FOS/MMP2/MMP9/MAPK1/IL10/JUN/IL6/CASP3/SOD1/HIF1A/DUOX2/NOS3/HSPB1/MPO/GSTP1/NFE2L2/NQO1/PARP1/CHUK	21
BP	GO:1903409	reactive oxygen species biosynthetic process	2.11757E-17	1.13219E-14	NOS2/PTGS2/IL10/SOD1/CYP1A2/CAV1/CYP1A1/ICAM1/IL1B/DUOX2/NOS3/IFNG/MPO/NQO1	14
BP	GO:2001233	regulation of apoptotic signaling pathway	5.20947E-16	2.38743E-13	AR/PTGS2/BAX/MMP9/RB1/TP63/CASP8/SOD1/HIF1A/CAV1/ICAM1/IL1B/NOS3/HSPB1/SERPINE1/IL1A/GSTP1/NFE2L2/PARP1	19
BP	GO:0034599	cellular response to oxidative stress	1.06893E-15	4.2864E-13	FOS/MMP2/MMP9/MAPK1/IL10/JUN/IL6/SOD1/HIF1A/NOS3/HSPB1/MPO/GSTP1/NFE2L2/NQO1/PARP1/CHUK	17
BP	GO:0007568	aging	2.94133E-15	1.04842E-12	PTGS2/FOS/CDKN1A/CASP9/MAPK1/IL10/JUN/TP63/SOD1/CYP1A1/ICAM1/VCAM1/SERPINE1/MPO/NFE2L2/NQO1/CHEK1	17
BP	GO:0097191	extrinsic apoptotic signaling pathway	4.84538E-15	1.5544E-12	AR/BAX/CASP9/CASP3/CASP8/CAV1/ICAM1/IL1B/NOS3/IL2/SERPI	15

					NE1/IFNG/IL1A/GSTP1/ERBB3	
BP	GO:2001234	negative regulation of apoptotic signaling pathway	7.17247E-15	2.09175E-12	AR/PTGS2/BAX/MMP9/RB1/CASP8/HIF1A/ICAM1/IL1B/NOS3/HSPB1/SERPINE1/IL1A/GSTP1/NFE2L2	15
BP	GO:0000302	response to reactive oxygen species	8.1543E-15	2.17992E-12	FOS/MMP2/MMP9/MAPK1/IL10/JUN/IL6/CASP3/SOD1/NOS3/MPO/GSTP1/NFE2L2/NQO1/CHUK	15
BP	GO:0048545	response to steroid hormone	5.82362E-14	1.34375E-11	AR/PTGS2/PPARG/FOS/CDKN1A/CASP9/IL10/RB1/IL6/CASP3/TP63/CAV1/ICAM1/GSTP1/PARP1/CLDN4/SPP1	17
BP	GO:0034614	cellular response to reactive oxygen species	5.86426E-14	1.34375E-11	FOS/MMP2/MMP9/MAPK1/IL10/JUN/IL6/SOD1/NOS3/MPO/NFE2L2/NQO1/CHUK	13
BP	GO:0046677	response to antibiotic	7.29367E-14	1.55987E-11	CASP9/IL10/JUN/IL6/CASP3/CASP8/SOD1/CYP1A1/ICAM1/VCAM1/IL2/GSTP1/NFE2L2/NQO1/AHR/CHUK	16
BP	GO:0070482	response to oxygen levels	8.48267E-14	1.70078E-11	NOS2/PTGS2/SLC6A4/PPARG/CDKN1A/PLAU/MMP2/CASP3/HIF1A/CAV1/MYC/CYP1A1/ICAM1/VCAM1/CCNB1/PLAT/NFE2L2	17
BP	GO:0044706	multi-multicellular organism process	9.90476E-14	1.86909E-11	AR/PTGS2/SLC6A4/FOS/MMP2/MMP9/MAPK1/SO	14

					D1/GJA1/CYP1A1/IL1B/THBD/CLDN4/SPP1	
BP	GO:1903034	regulation of response to wounding	1.3348E-13	2.37891E-11	CDKN1A/PLAU/IL10/CAV1/F3/GJA1/DUOX2/NOS3/PLAT/THBD/SERPINE1/NFE2L2/SPP1	13
BP	GO:0070997	neuron death	1.90413E-13	3.21497E-11	FOS/BAX/CASP9/IL10/RB1/JUN/CASP3/TP63/CASP8/SOD1/HIF1A/CCL2/IFNG/NQO1/PARP1/ERBB3	16
BP	GO:0071248	cellular response to metal ion	2.8821E-13	4.62288E-11	PTGS2/FOS/MMP9/MAPK1/JUN/SOD1/CYP1A2/CYP1A1/CCNB1/NFE2L2/NQO1/PARP1/CHUK	13
MF	GO:0005125	cytokine activity	1.13635E-09	2.1131E-07	IL10/IL6/IL1B/CCL2/CXCL8/IL2/IFNG/IL1A/CXCL2/SPP1/CD40LG	11
MF	GO:0005126	cytokine receptor binding	1.38111E-09	2.1131E-07	IL10/IL6/CASP3/CASP8/IL1B/CCL2/CXCL8/IL2/IFNG/IL1A/CXCL2/CD40LG	12
MF	GO:0020037	heme binding	3.33711E-09	3.40386E-07	NOS2/PTGS1/PTGS2/CYP3A4/CYP1A2/CYP1A1/DUOX2/NOS3/MPO	9
MF	GO:0046906	tetrapyrrole binding	6.26975E-09	4.79636E-07	NOS2/PTGS1/PTGS2/CYP3A4/CYP1A2/CYP1A1/DUOX2/NOS3/MPO	9
MF	GO:0016209	antioxidant activity	5.16321E-08	2.71121E-06	PTGS1/PTGS2/SOD1/DUOX2/MPO/GSTP1/NQO1	7

MF	GO:0048018	receptor ligand activity	5.3161E-08	2.71121E-06	IL10/EGF/IL6/IL1B/CCL2/CXCL8/IL2/IFNG/IL1A/CXCL2/SPP1/IGF2/CD40LG	13
MF	GO:0019207	kinase regulator activity	1.3701E-07	5.98929E-06	CDKN1A/EGF/CASP3/HSPB1/IL2/CCNB1/GSTP1/IGF2/ERBB3	9
MF	GO:0033613	activating transcription factor binding	1.14549E-06	4.17359E-05	PPARG/FOS/RB1/JUN/MYC/NFE2L2	6
MF	GO:0019209	kinase activator activity	1.22753E-06	4.17359E-05	CDKN1A/EGF/IL2/CCNB1/IGF2/ERBB3	6
MF	GO:0004601	peroxidase activity	2.02229E-06	6.1882E-05	PTGS1/PTGS2/DUOX2/MPO/GSTP1	5
MF	GO:0001085	RNA polymerase II transcription factor binding	2.87019E-06	7.48563E-05	AR/PPARG/FOS/RB1/JUN/NFE2L2/AHR	7
MF	GO:0016684	oxidoreductase activity, acting on peroxide as acceptor	2.93554E-06	7.48563E-05	PTGS1/PTGS2/DUOX2/MPO/GSTP1	5
MF	GO:0016705	oxidoreductase activity, acting on paired donors, with incorporation or reduction of molecular oxygen	3.39926E-06	8.00135E-05	NOS2/PTGS1/PTGS2/CYP3A4/CYP1A2/CYP1A1/NOS3	7
MF	GO:0019887	protein kinase regulator activity	7.6998E-06	0.000168296	CDKN1A/EGF/CASP3/HSPB1/CCNB1/IGF2/ERBB3	7
MF	GO:0070851	growth factor receptor binding	1.61127E-05	0.000309522	IL10/EGF/IL6/IL1B/IL2/IL1A	6
MF	GO:0030295	protein kinase activator activity	1.7148E-05	0.000309522	CDKN1A/EGF/CCNB1/IGF2/ERBB3	5
MF	GO:0016653	oxidoreductase activity, acting on NAD(P)H, heme protein as acceptor	1.71957E-05	0.000309522	NOS2/NOS3/NQO1	3

MF	GO:0097153	cysteine-type endopeptidase activity involved in apoptotic process	2.71995E-05	0.000462391	CASP9/CASP3/CASP8	3
MF	GO:0044389	ubiquitin-like protein ligase binding	3.15757E-05	0.000508535	CDKN1A/RB1/JUN/NFKBIA/CASP8/HIF1A/CCNB1/ERBB3	8
MF	GO:0004252	serine-type endopeptidase activity	4.38376E-05	0.000669003	PLAU/MMP2/MMP9/MMP1/F3/PLAT	6
CC	GO:0045121	membrane raft	1.83193E-07	1.80944E-05	PTGS2/SLC6A4/MAPK1/CASP3/CASP8/CAV1/GJA1/ICAM1/SELE/NOS3	10
CC	GO:0098857	membrane microdomain	1.88644E-07	1.80944E-05	PTGS2/SLC6A4/MAPK1/CASP3/CASP8/CAV1/GJA1/ICAM1/SELE/NOS3	10
CC	GO:0098589	membrane region	2.66095E-07	1.80944E-05	PTGS2/SLC6A4/MAPK1/CASP3/CASP8/CAV1/GJA1/ICAM1/SELE/NOS3	10
CC	GO:0005901	caveola	1.02296E-05	0.00052171	PTGS2/MAPK1/CAV1/SELE/NOS3	5
CC	GO:0044853	plasma membrane raft	4.58569E-05	0.001870962	PTGS2/MAPK1/CAV1/SELE/NOS3	5
CC	GO:0034774	secretory granule lumen	0.000154712	0.00504158	MAPK1/EGF/SERPINE1/ALOX5/MPO/GSTP1/IGF2	7
CC	GO:0060205	cytoplasmic vesicle lumen	0.000212268	0.00504158	MAPK1/EGF/SERPINE1/ALOX5/MPO/GSTP1/IGF2	7
CC	GO:0031983	vesicle lumen	0.000216129	0.00504158	MAPK1/EGF/SERPINE1/ALOX5/MPO/GSTP1/IGF2	7
CC	GO:0120111	neuron projection cytoplasm	0.000239666	0.00504158	MAPK1/SOD1/HIF1A/HSPB1	4
CC	GO:0009925	basal plasma membrane	0.000247136	0.00504158	ERBB2/CLDN4/ERBB3	3

CC	GO:1902554	serine/threonine protein kinase complex	0.000286529	0.005175955	CDKN1A/RB1/CCNB1/CHUK	4
CC	GO:0090575	RNA polymerase II transcription factor complex	0.000304468	0.005175955	PPARG/FOS/RB1/JUN/HIF1A	5
CC	GO:0005667	transcription factor complex	0.000338251	0.005307942	PPARG/FOS/RB1/JUN/HIF1A/PARP1/AHR	7
CC	GO:0045177	apical part of cell	0.000458493	0.006316373	ERBB2/GJA1/VCAM1/DUOX2/PLAT/CLDN4/ERBB3	7
CC	GO:0000307	cyclin-dependent protein kinase holoenzyme complex	0.000464439	0.006316373	CDKN1A/RB1/CCNB1	3
CC	GO:0005925	focal adhesion	0.000628868	0.007444285	SLC6A4/PLAU/MAPK1/CAV1/GJA1/ICAM1/HSPB1	7
CC	GO:1902911	protein kinase complex	0.000645854	0.007444285	CDKN1A/RB1/CCNB1/CHUK	4
CC	GO:0005924	cell-substrate adherens junction	0.000656849	0.007444285	SLC6A4/PLAU/MAPK1/CAV1/GJA1/ICAM1/HSPB1	7
CC	GO:0030055	cell-substrate junction	0.000695688	0.007469493	SLC6A4/PLAU/MAPK1/CAV1/GJA1/ICAM1/HSPB1	7
CC	GO:0044798	nuclear transcription factor complex	0.000790564	0.007996814	PPARG/FOS/RB1/JUN/HIF1A	5

Supplementary Table 7: The first 20 pathways of 53 common genes between drug and disease.

ID	Description	pvalue	p.adjust	geneID	Count
hsa05418	Fluid shear stress and atherosclerosis	1.07916E-19	2.21228E-17	2353/4313/4318/3725/857/3383/3553/6347/6401/7412/4846/5327/7056/3458/3552/2950/4780/1728/1147/2944	20
hsa04933	AGE-RAGE signaling pathway in diabetic complications	4.51516E-18	4.62804E-16	581/4313/5594/3725/3569/836/2152/3383/3553/6347/6401/7412/3576/4846/7056/5054/3552	17
hsa04657	IL-17 signaling pathway	4.7477E-17	3.24426E-15	5743/2353/4318/5594/3725/3569/836/4792/841/4312/3553/6347/3576/3458/2920/1147	16
hsa04668	TNF signaling pathway	8.77351E-16	4.49642E-14	5743/2353/4318/5594/3725/3569/836/4792/841/3383/3553/6347/6401/7412/2920/1147	16
hsa05142	Chagas disease	4.93639E-15	2.02392E-13	4843/2353/5594/3586/3725/3569/4792/841/3553/6347/3576/3558/5054/3458/1147	15
hsa05167	Kaposi sarcoma-associated herpesvirus infection	1.67375E-14	5.71866E-13	5743/2353/1026/581/842/5594/5925/3725/3569/836/4792/841/3091/4609/3383/3576/2920/1147	18
hsa05219	Bladder cancer	2.44534E-14	7.16135E-13	1026/4313/4318/5594/1950/5925/4312/2064/4609/3576/11186	11
hsa05161	Hepatitis B	3.34517E-13	8.572E-12	2353/1026/581/842/4318/5594/5925/3725/3569/836/4792/841/4609/3576/332/1147	16
hsa05215	Prostate cancer	1.34118E-12	3.0549E-11	367/1026/842/5328/4318/5594/1950/5925/4792/2064/5327/2950/1147	13

hsa05144	Malaria	9.93186E-12	2.03603E-10	3586/3569/3383/3553/6347/6401/7412/3576/3458/959	10
hsa05164	Influenza A	1.54118E-10	2.8722E-09	581/842/5594/3569/836/4792/841/3383/3553/6347/3576/3458/3552/1147	14
hsa05323	Rheumatoid arthritis	3.31702E-10	5.66657E-09	2353/3725/3569/4312/3383/3553/6347/3576/3458/3552/2920	11
hsa01524	Platinum drug resistance	5.13423E-10	8.09629E-09	1026/581/842/5594/836/841/2064/332/2950/2944	10
hsa05163	Human cytomegalovirus infection	5.77072E-10	8.25892E-09	5743/1026/581/842/5594/5925/3569/836/4792/841/4609/3553/6347/3576/1147	15
hsa05160	Hepatitis C	6.04311E-10	8.25892E-09	1026/581/842/5594/1950/5925/836/4792/841/4609/3458/1364/1147	13
hsa05133	Pertussis	7.72756E-10	9.90093E-09	4843/2353/5594/3586/3725/3569/836/3553/3576/3552	10
hsa05140	Leishmaniasis	8.82044E-10	1.06364E-08	4843/5743/2353/5594/3586/3725/4792/3553/3458/3552	10
hsa04064	NF-kappa B signaling pathway	1.12625E-09	1.28267E-08	5743/5328/4792/3383/3553/7412/3576/142/2920/1147/959	11
hsa04659	Th17 cell differentiation	1.53299E-09	1.65402E-08	2353/5594/3725/3569/4792/3091/3553/3558/3458/196/1147	11
hsa05162	Measles	1.97613E-09	2.02553E-08	2353/581/842/3725/3569/836/4792/841/3553/3558/3552/1147	12
